# Adaptive adjustment of profile HMM significance thresholds improves functional and metabolic insights into microbial genomes

**DOI:** 10.1093/bioadv/vbaf039

**Published:** 2025-03-21

**Authors:** Kathryn Kananen, Iva Veseli, Christian J Quiles Pérez, Samuel E Miller, A Murat Eren, Patrick H Bradley

**Affiliations:** Department of Microbiology, The Ohio State University, Columbus, OH 43210, United States; Helmholtz Institute for Functional Marine Biodiversity, 26129 Oldenburg, Germany; Alfred Wegener Institute, Helmholtz Centre for Polar and Marine Research, 27570 Bremerhaven, Germany; Department of Microbiology, The Ohio State University, Columbus, OH 43210, United States; Josephine Bay Paul Center for Comparative Molecular Biology and Evolution, Marine Biological Laboratory, Woods Hole, MA 02543, United States; Helmholtz Institute for Functional Marine Biodiversity, 26129 Oldenburg, Germany; Alfred Wegener Institute, Helmholtz Centre for Polar and Marine Research, 27570 Bremerhaven, Germany; Josephine Bay Paul Center for Comparative Molecular Biology and Evolution, Marine Biological Laboratory, Woods Hole, MA 02543, United States; Institute for Chemistry and Biology of the Marine Environment, University of Oldenburg, 26129 Oldenburg, Germany; Marine ‘Omics Bridging Group, Max Planck Institute for Marine Microbiology, 28359 Bremen, Germany; Department of Microbiology, The Ohio State University, Columbus, OH 43210, United States; Infectious Diseases Institute, The Ohio State University, Columbus, OH 43210, United States; Center of Microbiome Science, The Ohio State University, Columbus, OH 43210, United States

## Abstract

**Motivation:**

Gene function annotation in microbial genomes and metagenomes is a fundamental *in silico* first step toward understanding metabolic potential and determinants of fitness. The Kyoto Encyclopedia of Genes and Genomes publishes a curated list of profile hidden Markov models to identify orthologous gene families (KOfams) with roles in metabolism. However, the computational tools that rely upon KOfams yield different annotations for the same set of genomes, leading to different downstream biological inferences.

**Results:**

Here, we apply three open-source software tools that can annotate KOfams to genomes of phylogenetically diverse bacterial families from host-associated and free-living biomes. We use multiple computational approaches to benchmark these methods and investigate individual case studies where they differ. Our results show that despite their fundamental similarities, these methods have different annotation rates and quality. In particular, a method that adaptively tunes sequence similarity thresholds substantially improves sensitivity while maintaining high accuracy. We observe particularly large improvements for protein families with few reference sequences, or when annotating genomes from nonmodel organisms (such as gut-dwelling *Lachnospiraceae*). Our findings show that small improvements in annotation workflows can maximize the utility of existing databases and meaningfully improve *in silico* characterizations of microbial metabolism.

**Availability and implementation:**

Anvi’o is available at https://anvio.org under the GNU GPL license. Scripts and workflow are available at https://github.com/pbradleylab/2023-anvio-comparison under the MIT license.

## 1 Introduction

Metabolism, which links biochemical transformations to the survival and proliferation of organisms, is key to understanding microbial ecology and evolution. One important way to derive insights about microbial metabolism at scale is through (meta)genome annotation. Modern annotation tools can leverage decades of labor-intensive, mechanistic work on gene function to make high-throughput *de novo* inferences about metabolic potential. However, some of the most accurate methods, which use phylogenetic information and/or predictions of protein structure to find orthologs of genes of known function, are computationally demanding and scale poorly to increasingly large ‘omics surveys. Sequence homology searches offer a more scalable and streamlined approach to annotation. However, even when the same reference databases and alignment methods are used, implementation details can affect the rate of gene function recovery.

In this study, we focus on three methods that all rely on the commonly used Kyoto Encyclopedia of Genes and Genomes (KEGG) KOfam database of profile hidden Markov models (pHMMs) (Aramaki *et al.* 2020), yet process alignment scores differently to produce annotations: Kofamscan (Aramaki *et al.* 2020), MicrobeAnnotator (Ruiz-Perez *et al.* 2021), and anvi’o (Eren *et al.* 2021). All three tools begin by aligning open-reading frames (ORFs) to KOfam pHMMs  (Eddy 2011) and then applying a bit score threshold. For all pHMMs based on at least three sequences, KOfam provides such a threshold derived from cross-validation. The three methods then diverge in two main ways. First, some ORFs have no matches above the cutoff: Kofamscan by default discards these. In contrast, to catch homologs that may have missed this cutoff, anvi’o applies a second filter where the threshold is lowered by 50% (by default), then tests whether all matches passing both this filter and a global *e*-value threshold are from the same KOfam (Supplementary Fig. S1). MicrobeAnnotator can annotate additional homologs by BLASTing ORFs against a second database (SwissProt), transferring top KO annotations above a global threshold. Second, some KOfams lack a cross-validation threshold (we call these “nt-KOs”, short for “no threshold”). By default, neither Kofamscan nor anvi’o attempts to annotate nt-KOs. However, anvi’o can optionally apply a conservative adaptive threshold, based on the lowest bit score from sequences used to build the pHMM for each nt-KO. MicrobeAnnotator takes a more permissive approach, treating any match to nt-KOs as legitimate, regardless of bit score or *e*-value. A full explanation can be found in the Supplementary Methods.

The aims of this study are to conduct systematic benchmarks of annotation quality for these three pHMM-based methods, to investigate specific examples in detail, and to finally examine how differences in annotation affect downstream inferences about metabolism. We have chosen these methods because they differ only in how they process alignment scores obtained from the same set of pHMMs, enabling us to isolate the effects of the heuristics used by anvi’o and MicrobeAnnotator. In conducting these benchmarks, we are particularly interested in how these methods perform across a broad phylogenetic range of bacteria, including taxa that are not closely related to model systems, yet play important roles in ecosystems and human health. We hypothesize that heuristics to identify additional homologs could be most useful in these nonmodel clades, because their protein sequences may be more diverged from and less well represented among the sequences used to build the pHMM models. As there is much less direct experimental evidence that could be used to assign gene function in these clades (e.g. because they are currently genetically intractable), we instead evaluate annotation quality using multiple, complementary computational approaches. While the heuristics we investigate here could be applied to other sets of pHMMs, a benefit of focusing on the KOfam database is that it is a publicly available resource that is already widely used in microbial genomics. This means that methods for improving KOfam annotations can be incorporated into open source pipelines and have the potential for a broader impact on the field.

## 2 Methods

### 2.1 Databases

GTDB v214 (Parks *et al.* 2022) was used for genome data and taxonomic classification. Databases used by methods include KOfam (downloaded on 26 March 2024) (Aramaki *et al.* 2020), UniProt Swissprot (26 March 2024) (UniProt Consortium 2023), RefSeq (26 March 2024) (O’Leary *et al.* 2016), and Trembl (26 March 2024) (Boeckmann *et al.* 2003). EggNOG-mapper v5.0 (Huerta-Cepas *et al.* 2019, Cantalapiedra *et al.* 2021) and its accompanying database, as well as the NCBI Conserved Domain Database (CDD, scope B, version 26 March 2024), were used for the independent validation of annotations.

### 2.2 Resources and environment

Mambaforge v1.5.8 was used for package management. Snakemake v7.30.1 (Mölder *et al.* 2021) was used for workflow management. Eido v0.2.2 and Peppy v0.35.7 (Sheffield *et al.* 2021) were used to generate sample input tables and run the analysis as a Portable Encapsulated Project (PEP), a standardized way of organizing and describing input samples and their metadata which serves to increase the reproducibility of workflows during collaborative development. High-performance computing resources were supplied by the College of Arts and Sciences at The Ohio State University.

### 2.3 Sample selection

Potential genomes were randomly subsampled without replacement (*n* = 36) per selected family from GTDB for 11 families from different biomes (Parks *et al.* 2018, Parks *et al.* 2020, Rinke *et al.* 2021, Parks *et al.* 2022). To avoid including low-quality genomes in the analysis, we retained only genomes with publicly available annotations, as these tended to have higher CheckM completeness, lower CheckM contamination, and longer N50s. Files were retrieved with ncbi-genome-download v0.3.3 (Blin 2023). Prodigal v2.6.3 (Hyatt *et al.* 2010) was used to standardize gene calls across all tools and genomes. Anvi’o v8-dev (commit 545ba63) (Eren *et al.* 2021) was used to generate protein FASTA sequences for each genome.

### 2.4 Profile HMM methods

We used the profile HMM methods Kofamscan, anvi’o, and MicrobeAnnotator. Kofamscan v1.3.0 (Aramaki *et al.* 2020) was run twice, once using default settings and once with a threshold scale of 0.5 and an *e*-value threshold of 1×10^−5^ to match the settings used by the anvi’o bit score heuristic. Note that the latter settings in Kofamscan are less conservative than the bit score heuristic in anvi’o, because Kofamscan will keep all annotations with bit scores and *e*-values above the respective thresholds even when multiple distinct KOs match to a given gene, while the anvi’o heuristic does not include such annotations unless the additional hits are to the same KO. Running Kofamscan with these more relaxed parameters led to the recovery of an additional 34% more annotated genes per genome, on average; however, this increase drops to 10% when only annotations confirmed by EggNOG-mapper are considered.

We used MicrobeAnnotator v2.05 (Ruiz-Perez *et al.* 2021) (commit 9bbc5f6). Internal databases were retrieved and indexed using built-in scripts. Finally, KO prediction was performed twice: once using default settings, and once with the “—refine” flag.

Anvi’o v8-dev (commit 0e01dd7) (Eren *et al.* 2021) was initially run using default settings, which include the use of the adaptive bit score heuristic. To verify that the heuristic was the only difference from Kofamscan, it was run with the heuristic turned off, using the “—skip-bitscore-heuristic” flag. Finally, anvi’o was run with the “—include-stray-Kos” flag to incorporate annotations for nt-KOs.

### 2.5 Validation of annotations

All genomes were run through EggNOG-mapper 5.0 in DIAMOND mode (Huerta-Cepas *et al.* 2019, Cantalapiedra *et al.* 2021) and the resulting KO predictions were compared to KOs recovered from each profile HMM method. Annotations that matched at least one KO transferred via EggNOG-mapper were considered to be confirmed. In >90% of cases, EggNOG-mapper returned only zero or one KO. Because unconfirmed annotations include protein families that had no KO annotations in the EggNOG database, we also specifically counted cases where EggNOG-mapper had at least one KO annotation, but disagreed (in all cases, if more than one KO was found) with the KOs called by the profile HMM methods.

To determine how much these annotations disagreed in terms of function, we used the KEGG BRITE hierarchy (downloaded in JSON format, 21 November 2024) as well as Enzyme Commission (EC) numbers. We counted a match for the KEGG BRITE analysis if the KO annotated by a pHMM method shared all of its KEGG BRITE classifications, considering only the fourth, most specific level of the hierarchy, with at least one KO found by EggNOG-mapper. Note that when evaluating matches for KOs classified into multiple KEGG BRITE categories, we required that every category found in the profile HMM KO be found in the EggNOG-mapper KO and vice versa (i.e. one could not contain additional annotations). EC numbers for each KO were parsed from the KEGG Orthology descriptions (downloaded in TSV format, 17 November 2024). We counted a match if the EC number for the KO annotated by a pHMM method exactly matched one of the EC number(s) found by EggNOG-mapper (i.e. at all four levels). We excluded any EC numbers with dashes.

For the analysis of the top 20 genes in *Lachnospiraceae* with the most additional anvi’o or MicrobeAnnotator annotations, we exported the protein sequences in FASTA format, aligned them using Clustal Omega (Sievers *et al.* 2011, Sievers and Higgins 2018) with default settings, and constructed gene trees using FastTree 2 (Price *et al.* 2010). We also performed RPS-BLAST against the NCBI Conserved Domain Database (Marchler-Bauer *et al.* 2002, Lu *et al.* 2020, Wang *et al.* 2023) and kept the best annotation per sequence. For the glutaconyl-CoA decarboxylase and metallo-β-lactamase examples, manually selected sequences were downloaded from the KEGG GENES database and added to the alignment and the resulting tree. The HemNZ subfamily NCBI sequence identifiers were retrieved from the publication’s supplementary material (excluding eukaryotic sequences) (Cheng *et al.* 2022), and these were then run through the online NCBI CD-Search tool in batch mode (Yang *et al.* 2020). To examine the taxonomic distribution of the K01647 and K02495 sequences with different CDD annotations, we ran “tblastn” (Altschul *et al.* 1990) of the amino acid sequences from *Lachnospiraceae* genomes that were annotated with these KOs against all genomes in GTDB v214 (Parks *et al.* 2022) and kept all hits with *e*-value < 1*e*-15. For each set of query sequences with a given CDD annotation, we counted the number of genomes in each bacterial or archaeal family that had at least one hit from the query set.

In the correlation analysis for putative citrate synthases in *Lachnospiraceae*, anvi’o-annotated KOfam copy number profiles were assembled across all 36 *Lachnospiraceae* genomes, and Pearson correlation values were then calculated for each pair of distinct KOfams. The resulting full set of pairwise *P*-values was corrected for multiple testing using Storey’s *q*-value approach (Storey *et al.* 2015). Finally, for each of three queries (K01647, K01643, K05942), the KOfams with the top 10 and bottom 10 correlation values were reported, along with their raw *P*-value and corrected *q*-value (Supplementary Table S9).

Custom scripts were used to identify and analyze modules encoded in conserved gene neighborhoods that were more complete using anvi’o annotations. Briefly, we considered a module to be encoded in a gene neighborhood if over 50% of its annotated genes were no >5 gene calls apart from the previous and subsequent genes belonging to the module. If a module was encoded in a gene neighborhood in >30 genomes, we considered its gene neighborhood to be conserved. We then filtered these modules to include those that were more complete using anvi’o annotations compared to using either Kofamscan or MicrobeAnnotator annotations, and manually examined several examples to confirm that the additional annotations identified by anvi’o were part of the conserved gene neighborhood. When one of these additional annotations was not part of the conserved gene neighborhood in the particular genome we had selected, we used MUSCLE (Edgar 2004) to align several genes with this annotation to confirm that the sequences only annotated by anvi’o were similar to those annotated by all tools.

### 2.6 Pathway analysis

**Figure 1.**
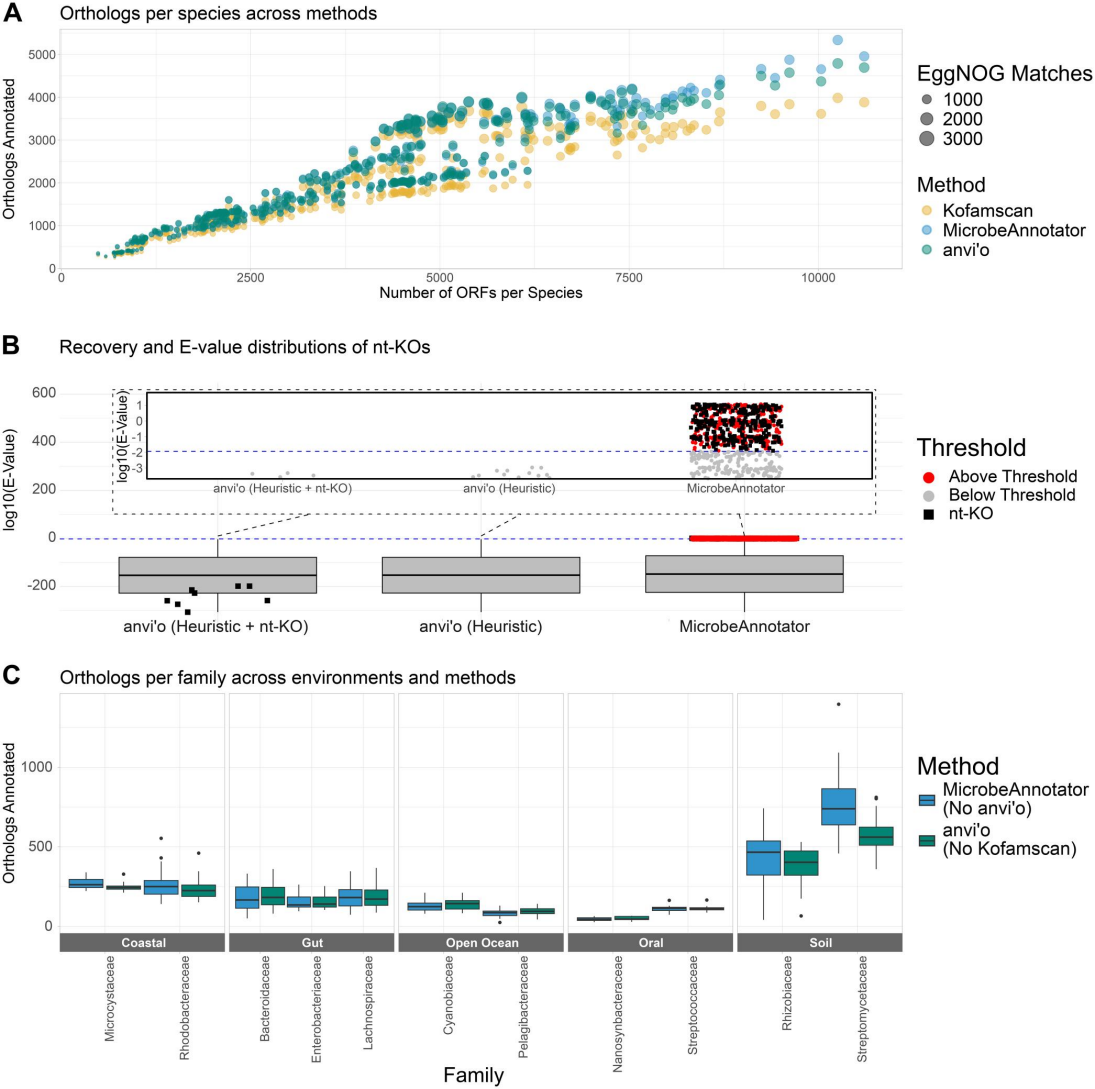
Heuristics consistently improve annotation rate. (A) Comparison of the total KOs identified by each tool with default parameters. The *x*-axis displays the number of open-reading frames (ORFs) in the genome. The size of each point corresponds to the number of orthologs that were also found by EggNOG-mapper as further evidence of correct KO annotation. (B) Distribution of *e*-values for KOs and nt-KOs as annotated by anvi’o or MicrobeAnnotator. The standard *e*-value threshold of log10(0.01) is indicated with the dashed blue line. The plot includes all unique *e*-value scores from each respective set of KO annotations from anvi’o run with default parameters, anvi’o run with the “—include-stray-Kos” flag, and MicrobeAnnotator run with default parameters. The inline plot shows only *e*-values above the all unique *e*-value scores above the threshold. The black points identify nt-KOs. (C) KOs that are uniquely identified by only one annotation method in each microbial family. Very few orthologs were identified by both anvi’o and MicrobeAnnotator but not Kofamscan (median was 1 per family).

In-house scripts were used to generate enzyme-txt files (https://anvio.org/help/8/artifacts/enzymes-txt/) to use as input for the anvi-estimate-metabolism program in anvi’o (https://anvio.org/help/8/programs/anvi-estimate-metabolism/) for each tool/parameter set combination. For anvi’o and Kofamscan annotations, we included in the enzyme-txt files only the hit with the lowest *e*-value for each gene to match the behavior of MicrobeAnnotator. Per-pathway completeness scores for each metabolic pathway in the KEGG MODULE database were generated with the anvi-estimate-metabolism program. Individual pathway completeness scores were averaged across all genomes in each family per method with highest number of recovered KOs (see Fig. 1A). We created custom metabolic modules for butyrate biosynthesis from Acetyl-CoA by identifying the KOs associated with this pathway as described using Enzyme Commission (EC) numbers in (Vital *et al.* 2014, Louis and Flint 2017, Lin *et al.* 2024), with the addition of the crotonase enzyme K17865 (EC: 4.2.1.55) for the dehydration reaction from 3-hydroxybutyryl-CoA to crotonyl-CoA. One module (“BUTANOATE”) describes the full set of enzymes with all potential alternative KOs, another module (“SUBSPEC”) includes only the most substrate-specific enzymes whenever possible, and the last module (“BUCASYNOP”) includes only the 5-gene core operon for butyryl-CoA synthesis. After generating a user-defined modules database with “anvi-setup-user-modules,” we estimated pathwise completeness for these pathways for the default annotation modes of each tool within the set of 36 *Lachnospiraceae* genomes using “anvi-estimate-metabolism.”

### 2.7 Visualization

R version v4.3.2 and v4.4.0 (R Core Team 2024) was used to generate all graphics with the exception of Figs 3 and 4A; and Supplementary Figs S1, S5, and S10. Barplots were generated using tidyverse v2.0.0 (Wickham *et al.* 2019), plyr v1.8.9 (Wickham 2011), and ggplot2 v3.5.0 (Wickham 2016). Scatterplots were generated with ggplot2. Heatmaps were generated using viridis v0.6.5 (Garnier *et al.* 2024), data.table v1.15.4 (Barrett *et al.* 2024), devtools v2.4.5 (Wickham *et al.* 2022), and the ComplexHeatmap v2.15.4 package (Gu *et al.* 2016, Gu 2022). Piecharts were generated with plotly v4.10.4 (Sievert 2019). In Fig. 4A, substrate-specific enzymes were identified as participating in only one reaction in KEGG, or had no reaction annotation in KEGG but performed a single reaction based on their EC number; these were marked in bold. Promiscuous enzymes were either annotated to multiple KEGG reactions, or were a general cofactor-metabolizing subunit (e.g. involved in electron transport) of a larger enzyme complex. In Fig. 4B, drug resistance modules were plotted for the three methods by subsetting any module category that contained the string “Drug resistance.” In Supplementary Fig. S7, per-genome, per-pathway completeness for anvi’o and MicrobeAnnotator over Kofamscan was calculated and graphed. For Supplementary Fig. S10A, the heatmap of completeness scores was generated with the anvi’o program “anvi-interactive.” For Supplementary Fig. S10B, we colored the corresponding KEGG pathway map for butanoate metabolism (accession ko00650) according to which tool(s) were able to annotate each enzyme in the map in at least one of the *Lachnospiraceae* genomes. To create the map figure, custom scripts were used to parse the annotation files, identify the set of tools annotating each KO, and use the resulting table to color the KEGG pathway map. For Fig. 3 and Supplementary Fig. S5, the gene neighborhood visualizations were exported from the “anvi-inspect” program in anvi’o, and the multiple sequence alignment was visualized with the Python library “pyMSAviz” (Shimoyama 2022). Trees were parsed and visualized with the R packages ape v5.8 (Paradis and Schliep 2019), phytools v2.1.1 (Revell 2024), ggtree v3.12.0 (Yu *et al.* 2017), and ggtreeExtra (Xu *et al.* 2021); multiple sequence alignments were loaded and visualized with Biostrings v2.72.0 (Pagès *et al.* 2024) and ggmsa v1.10.0 (Zhou *et al.* 2022). Supplementary Figure S11 was generated using gggenes v0.5.1 (Wilkins 2023). Other R packages used for reading, processing, or visualizing data included ggnewscale v0.5.0 (Campitelli 2024), ggeasy v0.1.5 (Carroll *et al.* 2024), patchwork v1.3.0.9000 (Pedersen 2024), tidyverse v2.0.0 (Wickham *et al.* 2019), jsonlite v.1.8.9 (Ooms 2014), and arrow v17.0.0.1 (Richardson *et al.* 2024).

## 3 Results and discussion

To systematically assess how these subtle methodological differences affect annotation quantity and quality, we used these three tools to annotate KEGG Orthologs (KOs) (Kanehisa *et al.* 2016) in a sample of 396 genomes from 11 bacterial families (Supplementary Table S1), representing five different biomes and ranging from clades that included common model organisms and pathogens (*Enterobacteriaceae*) to less-well-studied families (*Lachnospiraceae*, *Nanosynbacteriaceae*). We assessed annotation quality using three strategies: comparison to a phylogeny-based method, comparing quality scores and sequences, and evaluating whether these annotations made sense given genomic context. Finally, we used these annotations to estimate metabolic pathway completeness, and as a case study, explored different pathways for butyrate synthesis in the gut commensal *Lachnospiraceae*.

We observed large differences in annotation rate across all three tools. MicrobeAnnotator and anvi’o, which use additional heuristics for recovering homologs, consistently annotated more ORFs per genome compared to Kofamscan (Fig. 1A). Using the bit score heuristic in anvi’o enabled recovery of ∼13% more annotated genes per genome (range: 3.1%–21%, paired Wilcox test *P* < 2.2×10^−16^). Disabling this heuristic made the results identical to Kofamscan’s. We also ran MicrobeAnnotator in “refine” mode, which additionally maps genes against SwissProt and converts Enzyme Commission (EC) numbers and InterPro accessions to KO accessions; however, additional KOs recovered ultimately resulted in no additional unique annotations per genome, so we did not use this mode for comparisons moving forward.

Because MicrobeAnnotator always annotates nt-KOs, we ran an additional comparison enabling nt-KO annotations in anvi’o. This increased its annotation rate on average by 0.07% per genome (*P* < 2.2×10^−16^), yielding approximately 1536 ORFs with confirmed annotations, compared to 1457 from MicrobeAnnotator. Notably, for MicrobeAnnotator, annotation *e*-values repeatedly fell outside the standard cutoff of *e* < 0.01, with many surpassing 1 (Fig. 1B), indicating that nt-KOs annotated using anvi’o are more likely to be reliable.

To determine how annotation rates varied across bacterial taxa, we counted KOs annotated uniquely by MicrobeAnnotator or anvi’o (none were uniquely annotated by Kofamscan). Between 24 and 1396 orthologs were uniquely annotated by MicrobeAnnotator, and between 28 and 811 with anvi’o (Fig. 1C). *Enterobacteriaceae* genomes had fewer unique KOs, likely because this family contains *Escherichia coli* along with several common pathogens, and is expected to be well-represented in KEGG. In contrast, many unique annotations were recovered for *Rhizobiaceae*, a family containing numerous plant-associated microbes with 7K genomes in GTDB, compared to 97K for *Enterobacteriaceae*. For *Rhizobiaceae* and *Streptomycetaceae*, the high number of orthologs is likely due to the large mean genome size of the bacteria representing these families (Supplementary Table S2). All annotations unique to MicrobeAnnotator were nt-KOs.

### 3.1 Validation of annotation accuracy using multiple complementary methods

Evaluating the accuracy of the annotation results is a difficult yet critical step to contextualize the differential performance of the three tools. Though experimental characterization of gene function is the most reliable way to confirm annotations, doing so in a scalable manner for thousands of genes across hundreds of genomes would be challenging, especially in organisms that are not genetically tractable and/or have few cultured representatives. We therefore relied on three orthogonal yet complementary computational strategies to assess annotation quality: (i) comparing to a tool that uses local alignment to sets of orthologs, EggNOG-mapper; (ii) analyzing bit score distributions, multiple sequence alignments, and conserved domain (CD) annotations (Wang *et al.* 2023) of the most differentially annotated KOs in the *Lachnospiraceae* family; and finally, (iii) incorporating genomic context and gene synteny into our analyses to evaluate the completeness of metabolic modules encoded in conserved gene neighborhoods.

#### 3.1.1 Confirming annotations via local alignment to EggNOG orthologous groups

The tool EggNOG-mapper can transfer protein annotations, including of KOs, by performing a local alignment search with DIAMOND (Buchfink *et al.* 2021) against a fine-grained pre-computed ortholog database (EggNOG v5) (Cantalapiedra *et al.* 2021). EggNOG orthologous groups (OGs) are defined differently from KOs, and so we do not expect a one-to-one correspondence; one major difference is that KOs may include multiple isofunctional protein families (Kanehisa *et al.* 2016), while EggNOG OGs include tree information and prioritize phylogenetic coherence, aiming to capture sequences descending from a single speciation event. In prokaryotes, horizontal gene transfer is an important factor in gene family evolution (Koonin and Wolf 2008), which complicates the assumption in EggNOG that genes evolve primarily by vertical descent, duplication, and loss. That said, if KO annotations from the HMM-based methods match those transferred by EggNOG-mapper, this increases our confidence in the results.

Kofamscan returned the largest percentage of KO annotations across all genomes confirmed by EggNOG-mapper, with 84% (576 729/689 401 genes), followed by anvi’o at 79% (608 311/774 888 genes). MicrobeAnnotator recovered the smallest number (577 041/781 222 genes, 74%). However, anvi’o recovered the highest total number of confirmed annotations: compared to Kofamscan, 6.54% more confirmed annotations were recovered per genome on average using anvi’o (range: 1.2%–15%, *P* < 2.2×10^−16^), versus 0.65% for MicrobeAnnotator. When we only considered the additional annotations not found by Kofamscan, 37% of the anvi’o-specific annotations were confirmed by EggNOG-mapper, compared to only 0.3% of the MicrobeAnnotator-specific annotations. However, in the majority of cases that could not be confirmed, this was actually because the EggNOG OG had no KO annotations to transfer, so it is possible that these were false negatives on the part of EggNOG-mapper. To get a better sense of the false positive rate, we therefore focused on cases where EggNOG-mapper could annotate at least one KO, but where none of these annotations agreed with the profile HMM method. The fraction of annotations that disagreed with EggNOG was 3.5% for Kofamscan, 3.9% for anvi’o, and 7.2% for MicrobeAnnotator (Supplementary Fig. S2). When considering only annotations not made by Kofamscan as above, anvi’o disagreed with EggNOG-mapper 7.5% of the time, whereas MicrobeAnnotator disagreed 35% of the time.

Even when these KOfam annotations disagreed, they were sometimes still very close in terms of function. For example, anvi’o annotated one Nanosynbacteraceae gene as the KO K03587, which KEGG describes as “ftsI; cell division protein FtsI (penicillin-binding protein 3).” EggNOG-mapper annotated this same gene as K08384, or “spoVD; stage V sporulation protein D (sporulation-specific penicillin-binding protein)”; furthermore, the EggNOG-mapper free text description for this gene is actually “Cell division protein FtsI penicillin-binding protein 2.” This would have been scored as a false positive above. To assess this type of similarity systematically, we used the KEGG BRITE functional hierarchy, as well as Enzyme Commision (EC) numbers predicted either by EggNOG-mapper or from KOs. First, we counted cases where the KO predicted from a pHMM method had identical KEGG BRITE classifications to at least one KO transferred from EggNOG-mapper, even though the KOs themselves did not overlap. This was true in 28% of the Kofamscan cases that disagreed, 27% for anvi’o, and 18% for MicrobeAnnotator (when considering only the annotations unique to anvi’o or MicrobeAnnotator, we observed KEGG BRITE matches in 24% and 11% of cases, respectively). We next counted cases where despite no KO overlap, one of the EC numbers transferred using eggNOG-mapper exactly matched an EC number predicted from the KOs. This was true for Kofamscan 17.6% of the time, 17.4% for anvi’o, and 9.63% for MicrobeAnnotator (16.6% and 3.8% considering only annotations specific to these two tools, respectively).

In total, we consider three types of matches between EggNOG and the HMM-based annotation tools: (i) both tools directly predict one of the same KOs; (ii) an EC number from EggNOG agrees with an annotated KO at all four positions; and (iii) a KO predicted by one tool shares all its KEGG BRITE annotations with one from EggNOG. According to these criteria, when EggNOG makes at least one KO or EC prediction, Kofamscan annotations match 98.7% of the time. The “new” annotations made by anvi’o but not Kofamscan match 91.7% of the time, while the “new” annotations from MicrobeAnnotator match 38.1% of the time. Overall, these results suggest while both anvi’o and MicrobeAnnotator recover substantially more annotations than Kofamscan, only anvi’o is able to do so while maintaining a similar level of quality.

#### 3.1.2 Using bit score distributions and sequence analysis to assess conservation within KO groups

To investigate the validity of annotations identified via the bit score heuristic in anvi’o in more detail, we next analyzed the 20 KOs where the heuristic identified the most additional hits in the *Lachnospiraceae*. Overall, annotations to these 20 KOs in *Lachnospiraceae* tended to have moderately lower bit score distributions compared to annotations in other taxa, meaning that more *Lachnospiraceae* sequences dropped below the model’s threshold (Supplementary Fig. S3). This is what we would expect if *Lachnospiraceae* sequences were less well represented in the sequences used to train the KO pHMMs. We note that the same analysis could not be performed for MicrobeAnnotator because the 20 KOs with the most additional *Lachnospiraceae* annotations were all nt-KOs, and thus lacked thresholds. Multiple sequence alignments (MSAs) and gene trees generally showed high sequence conservation among the *Lachnospiraceae* proteins annotated to a given KO; furthermore, in many cases, proteins found only with the anvi’o heuristic clustered with proteins found without the heuristic (Supplementary Fig. S4).

**Figure 2.**
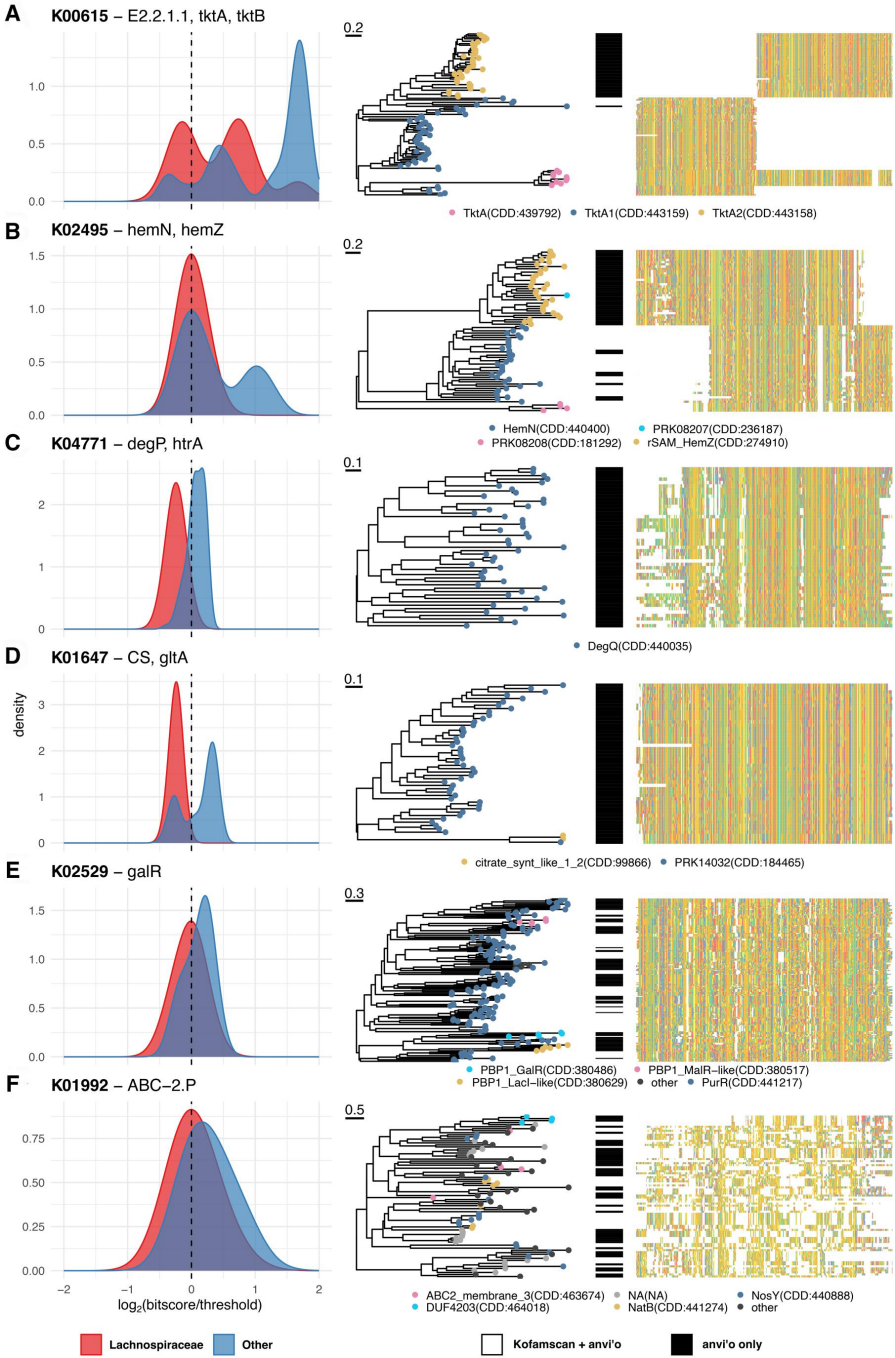
Bit score distributions, protein trees and multiple sequence alignments of selected differentially annotated *Lachnospiraceae* KOs. (A–F) Each panel shows one KO for which more sequences were annotated by anvi’o than by Kofamscan in *Lachnospiraceae* genomes. The density plots on the left show the log_2_-normalized distribution of the bit score ratio (bit score divided by the bit score threshold) across all annotations of the KO in *Lachnospiraceae* genomes (red) versus genomes from other bacterial families (blue). On the right, FastTree 2 (Price *et al.* 2010) trees with scale bars in substitutions per site show the relationship between protein sequences, and multiple sequence alignments allow visualization of conserved residues (Sievers *et al.* 2011, Sievers and Higgins 2018). Tree leaves are colored according to each sequence’s Conserved Domain annotation (Marchler-Bauer *et al.* 2002, Lu *et al.* 2020, Wang *et al.* 2023) and black bars indicate genes that were only annotated by anvi’o.

We did also observe some KOs where the anvi’o heuristic identified a sequence cluster in *Lachnospiraceae* that was not found using Kofamscan. We therefore further classified these sequences using RPS-BLAST and the curated NCBI Conserved Domain Database (CDD) (Marchler-Bauer *et al.* 2002, Lu *et al.* 2020, Wang *et al.* 2023), and also compared the results to annotations from the KEGG GENES database, which is the source of the sequences used to build the KO pHMMs. In the case of K00615 (transketolase; Fig. 2A), both anvi’o and Kofamscan identified sequences in the CD families TktA and TktA1 (transketolase 1 or TktA1), while only anvi’o identified sequences in the family TktA2. Transketolase can be encoded as either a single gene (TktA) or as two genes, corresponding to the N- and C-terminal domains of the protein (TktA1 and TktA2), as in the bacterium *Carboxydothermus hydrogenoformans* (James *et al.* 2020). Indeed, KEGG’s curated annotations for K00615 include the *C.hydrogenoformans* TktA1 and TktA2 sequences, validating these additional anvi’o annotations.

A similar pattern was observed for the KO K02495 (coproporphyrinogen III oxidase; Fig. 2B). Both tools recovered proteins in the HemN family, though anvi’o recovered more than Kofamscan; however, only anvi’o additionally found hits to the rSAM_HemZ family. HemZ and HemN are close homologs: both have coproporphyrinogen III oxidase activity, and both HemZ and HemN sequences were used to construct the K02495 protein family in KEGG. To identify other possible homologs, we separated the K02495 *Lachnospiraceae* sequences by their top CDD hit, then used BLAST (Altschul *et al.* 1990) to compare each group against all genomes in the Genome Taxonomy Database (GTDB) (Parks *et al.* 2022). Homologs of the sequences in PRK08208 and HemN (annotated by both Kofamscan and anvi’o) were broadly distributed, while the rSAM_HemZ and PRK08207 family sequences (annotated only by anvi’o) returned almost exclusively *Lachnospiraceae* genomes (Supplementary Fig. S13, Supplementary Table S7). Thus, anvi’o appears to be capturing a *Lachnospiraceae*-specific variant of these genes. The HemN/HemZ superfamily does contain some subfamilies with divergent functions. While experimental evidence would be required to unambiguously establish the function of the anvi’o-specific hits, we did additionally analyze a recently curated set of proteins that spanned known subfamilies (Cheng *et al.* 2022). We found that all of the curated HemZ proteins, and none of the bacterial proteins from other subfamilies, matched either the rSAM_HemZ or the PRK08207 families, bolstering our confidence that the additional anvi’o annotations are authentic HemZ sequences (Supplementary Table S8).

Some KOs, such as K04771 (degP/htrA serine proteases; Fig. 2C) and K01647 (citrate synthase; Fig. 2D), were missed entirely by Kofamscan. For K04771, all of the anvi’o hits fell into the DegQ CD family; DegQ is a close homolog of DegP that can complement a DegP deletion in *E.coli* (Waller and Sauer 1996), and the KO family K04771 includes both DegP and DegQ sequences. For K01647, all hits matched CD families in the citrate synthase/ATP-citrate lyase/citryl-CoA lyase superfamily (cl00416). All but two specifically matched the PRK14032 family best; these remaining two aligned best to a “citrate-synthase-like” family, but still second-best to PRK14032. As above, we performed a BLAST analysis of these matches against GTDB, and found that homologs to the PRK14032 matches were heavily enriched in *Lachnospiraceae* genomes, as well as in other commensal Clostridia such as the *Ruminococcaceae* and *Acutalibacteraceae.* Homologs to the “citrate-synthase-like” family hits were more broadly distributed, but also enriched in *Lachnospiraceae* (Supplementary Fig. S14).

Similarly to the case of HemN/HemZ above, no members of PRK14032 have yet been experimentally characterized, and the broader cl00416 superfamily (to which it belongs) also includes sequences annotated as citrate lyases and 2-methylcitrate synthases. We therefore sought further evidence for the specific function of these novel K01647 hits. We performed a correlation analysis of anvi’o-annotated KOfam copy numbers across the *Lachnospiraceae*, retaining the top 10 most (anti)-correlated accessions (Supplementary Table S9). In addition to K01647, we also considered two other KOfams that could play a similar role: K01643, a subunit of a citrate lyase/citrate CoA-transferase, and K05942, an *Re*-citrate synthase [typical citrate synthases have *Si* stereospecificity, but some Clostridia are known to have the phylogenetically unrelated *Re* version, potentially in addition to an *Si*-version (Li *et al.* 2007)].

We found that of the three, K01647 was the most positively correlated with isocitrate dehydrogenase (K00031, *r* = 0.57, *q* = 0.010). K05942 was correlated to a lesser extent (*r* = 0.35, *q* = 0.22), while K01643 was nonsignificantly anti-correlated (*r* = –0.22, *q* = 0.45). Notably, neither isocitrate dehydrogenase nor any close homolog plays a part in the 2-methylcitrate cycle (Brämer *et al.* 2002). Methylisocitrate is instead metabolized by a homolog of isocitrate lyase; neither isocitrate nor methylisocitrate lyases (K01637, K03417) were detected by anvi’o in the *Lachnospiraceae*. K01647 was also correlated with an NADH-quinone oxidoreductase involved in electron transport (K00335, *r* = 0.55, *q* = 0.014). In contrast, the citrate lyase subunit K01643 correlated best with the other subunits of citrate lyase (K01644 and K01646, *r* > 0.9, *q* = 9.1 × 10^−14^) and almost as well with carboxylic acid transport membrane proteins (K07793, K07795, K11690, K07794, *r* > 0.88, *q* < 1.2 × 10^−9^), suggesting a role in metabolizing extracellular tri- and/or di-carboxylic acids. *Re*-citrate synthases, interestingly, were anti-correlated with citrate lyase (*r* = –0.43, *q* = 0.010), and most positively correlated with genes having to do with pilus assembly and pseudaminic acid synthase (*r* = 0.49, *q* = 0.044), suggesting a possible connection to adherent growth (McKee *et al.* 2018).

Based on this analysis, we believe the novel K01647 hits are likely to be real *Si-*citrate synthases that were not detected simply because they came from an under-studied region of sequence space. That said, it is not always possible to confidently distinguish between citrate and 2-methylcitrate synthase activity by sequence alone, and many proteins have at least some activity for both substrates (Gerike *et al.* 1998). For this reason, without experimental evidence, we cannot exclude the possibility that these are 2-methylcitrate synthases or have some entirely novel functionality.

Finally, we also observed cases such as K02529 (galR/LacI family repressors; Fig. 2E) and K01992 (ABC-2 type prokaryotic permeases; Fig. 2F) where anvi’o-specific hits were scattered throughout the gene tree, indicating that they were closely related to the hits found by both methods. For K01992 in particular, we noted that although the most frequent CD family annotations included known ABC-2 permease families such as NosY, NatB, and ABC2_membrane_3, the sequences showed a particularly low degree of conservation, regardless of whether the annotations were made using anvi’o or Kofamscan. This is likely because of the high diversity of ABC-2 type permease sequences used to train the K01992 pHMM; indeed, the Kofamscan cross-validation bit score threshold for this family is only 18, much lower than for other KOs. Thus, while they tend to have lower bit scores overall (possibly because the *Lachnospiraceae* sequences are less-well represented in the training data), the novel anvi’o annotations in this family are consistent with Kofamscan and/or the KEGG GENES database, both in terms of sequence and function.

#### 3.1.3 Verifying tool-specific annotations using syntenic metabolic modules

**Figure 3.**
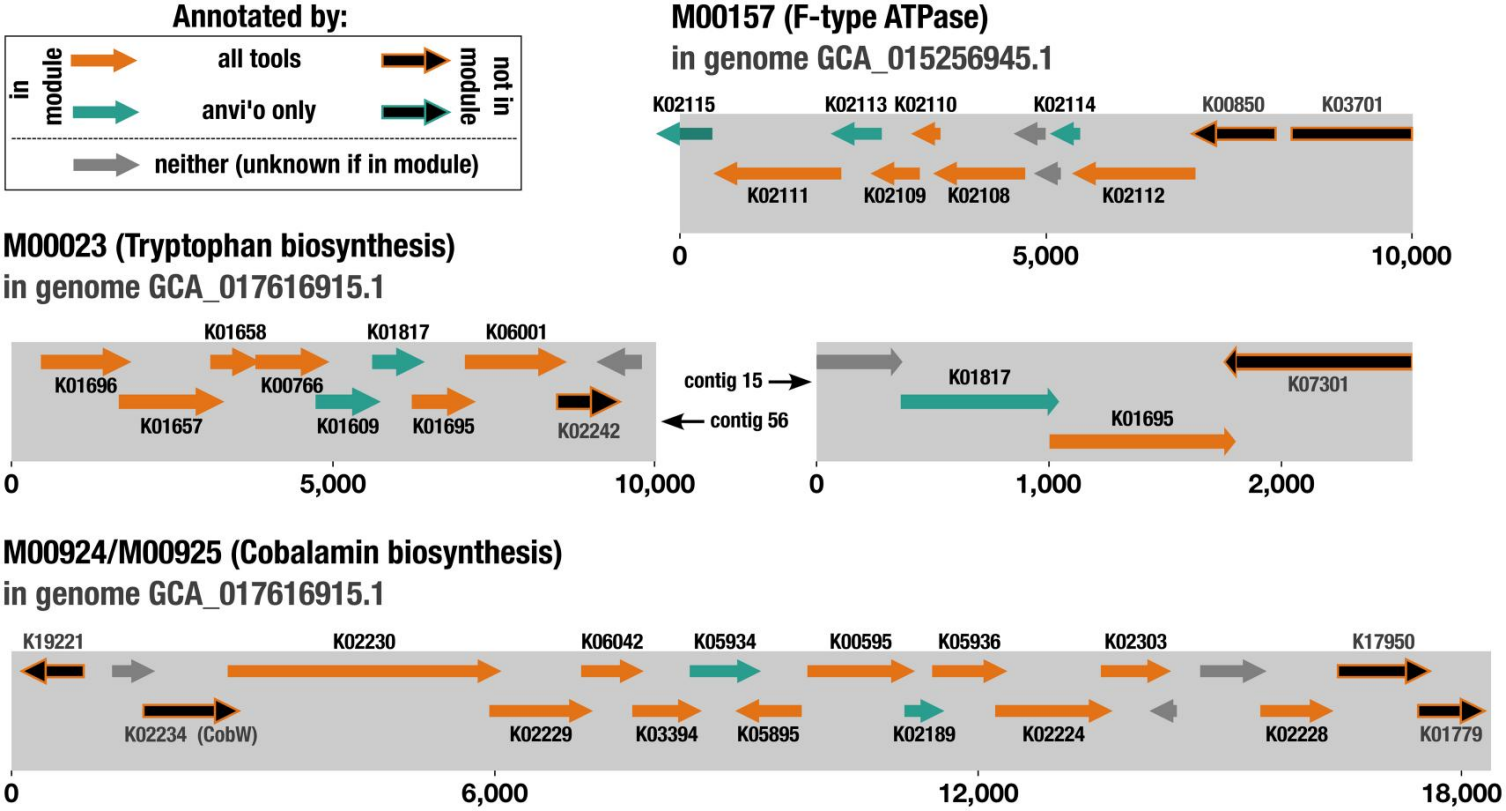
Examples of modules in operon-like structures that are more complete using anvi’o annotations. For each module, gene orders and lengths are shown as they appear in a single representative genome, but similar organization was seen across multiple genomes. Genes are shown as arrows that indicate whether they are on the forward or reverse strand. Each gene is colored or outlined according to which tool(s), if any, were able to annotate it in the displayed genome. Full-color arrows indicate genes belonging to the example module, black arrows indicate genes that do not belong to the module, and gray arrows indicate genes without any functional annotation. Additional examples are shown in Supplementary Fig. S5, and annotation counts for the KOs in these modules across all genomes can be found in Supplementary Table S3.

In bacteria, functions that participate in the same metabolic process are often encoded by genes that are close together on the chromosome, enabling yet another means to interpret the validity of additional annotations by considering the upstream and downstream functions in the larger genomic context (Overbeek *et al.* 1999, Galperin and Koonin 2000). To leverage these conserved gene neighborhoods as a validation strategy, we identified 143 metabolic modules in which the majority of component genes were annotated close together in the genomes of >30 bacterial species (out of the 396 species included in our analysis). 110 of these modules were more complete using anvi’o annotations compared to the other two tools in at least one genome. We then investigated several examples to determine whether the additional annotations made sense (i.e. were co-localized with other genes from the module). Indeed, in these cases, anvi’o identified additional annotations within the genomic neighborhood of the other genes in the module that were annotated by all tools (Fig. 3, Supplementary Fig. S5). For the KOs involved in these modules, on average, 6% of annotations were identified exclusively by anvi’o, while none were annotated exclusively by MicrobeAnnotator (Supplementary Table S3).

Overall, the above results suggest that the bit score heuristic in anvi’o allows up to 21% more genes to be annotated in microbial genomes. These genes have moderately diverged protein sequences compared to those from which the KOfams were built, yet the annotation accuracy is well-supported by our computational analysis, which includes independent sources of gene functions, sequence alignments, and the genomic contexts in which annotations appear.

### 3.2 Metabolic implications of annotation performance in downstream analyses

So far, we have demonstrated differences in functional annotation rate without exploring its implications on metabolic insights. We next assessed the downstream impacts of variable annotation recovery by calculating KEGG metabolic module completeness scores (Veseli *et al.* 2023) for all 11 families across all three tools. For each family, we also determined the 10 modules whose completeness scores differed most across tools (Supplementary Table S4). Overall, anvi’o resulted in 11% more modules per genome with ≥80% completeness compared to MicrobeAnnotator, and 12% more compared to Kofamscan. Averaging across all genomes in a family, median completeness scores increased in 15% of modules with anvi’o and in 13% with MicrobeAnnotator. To reduce the influence of single-gene modules reporting 100% completeness, we repeated the above analysis for only multi-gene modules. When considering only these modules, the difference in the number of complete modules increased slightly to 13% for anvi’o when compared to Kofamscan. For anvi’o, median completeness scores improved most often for less-well-studied families, such as *Nanosynbacteraceae* (higher than Kofamscan in 38% of cases), and least often for the well-studied *Enterobacteriaceae* (higher in 4.9% of cases). MicrobeAnnotator did not display this trend (Supplementary Tables S4 and S5, Supplementary Figs S6 and S7). Furthermore, at ≥80% completeness, anvi’o uniquely identified 142 modules at the species level (compared to 1544 modules identified by all tools).

**Figure 4.**
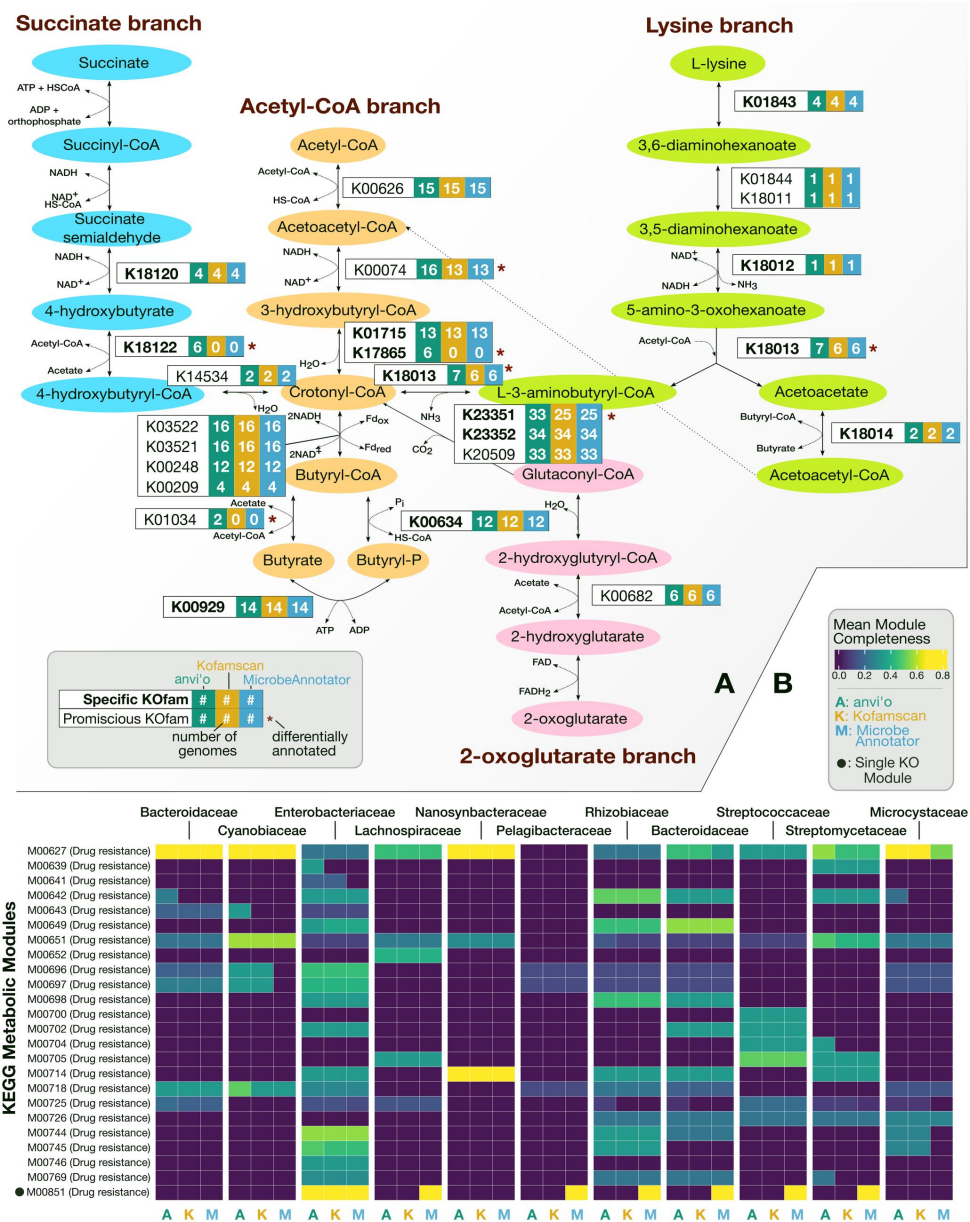
Impact of differential annotation performance on the downstream identification of metabolic pathways. (A) Annotated butyrate production pathways, demonstrating the number of genomes with annotation(s) for each enzyme from each of the three software tools (with default parameters). Pathways are based on Vital *et al.* (2014). Substrate-specific enzymes have bold labels, while those that are promiscuous are labeled in regular text. Asterisks indicate enzymes for which at least one of the three tools yielded different results. (B) Per-family mean completeness scores for all drug resistance modules in KEGG recovered by each method. Modules marked with circles indicate that the module consists of only one KO.

We did identify modules unique to MicrobeAnnotator, especially drug resistance pathways (Fig. 4B, Supplementary Fig. S8). However, these appeared unlikely given the biological context: annotations from MicrobeAnnotator suggest a complete carbapenem resistance pathway (M00851) in all families except *Synechococcaceae* and *Nanosynbacteraceae*, whereas anvi’o and Kofamscan only detect this in *Enterobacteriaceae*. While carbapenem resistance was previously reported in some marine microbiota (Dewi *et al.* 2021), to be found fully complete among all species in nearly all families is unlikely. Additionally, this module has only a single step in KEGG, meaning only one KO out of several alternatives is needed to consider it complete. By far, the most common KO that MicrobeAnnotator annotated in this module was the nt-KO K19099, a metallo-β-lactamase found in *P. aeruginosa* (Castanheira *et al.* 2004). When we performed a multiple sequence alignment including the MicrobeAnnotator hits and the two sequences used to build K19099, we found a low degree of sequence conservation (Supplementary Fig. S9). A Conserved Domain Database search revealed that these sequences were likely metallo-β-lactamases, but this is a very broad and functionally diverse group of enzymes, and most do not participate in carbapenem resistance. Thus, these carbapenem resistance annotations were likely driven by MicrobeAnnotator’s lenient approach to identifying nt-KOs.

As a specific test case, we next compared each tool’s ability to annotate enzymes involved in the biosynthesis of butyrate, a key metabolic product of gut microbes. This pathway, previously found to be complete in about 41% of *Lachnospiraceae* genomes, can terminate in two branches: butyryl-CoA transferase (BCoAT) or butyryl kinase (buk) (Lin *et al.* 2024). We curated a gene set for Clostridial butyrate production, and then computed completeness in 36 *Lachnospiraceae* genomes. While all three tools comparably annotated the trunk of the pathway from acetyl-CoA (Supplementary Fig. S10, Supplementary Table S6), anvi’o detected the KOfam for the second step (3-hydroxybutyryl-CoA dehydrogenase) in three additional genomes. Anvi’o also detected the BCoAT branch in two genomes, while the other tools found it in none (Fig. 4A). Furthermore, anvi’o exclusively detected two additional KOfams in six genomes each—K17865 in the acetyl-CoA branch, and K18122 in the succinate branch. With anvi’o, the pathway for acetyl-CoA to butyrate was >80% complete with at least 4/5 steps detectable in 33.3% of these genomes, in line with previous estimates (Lin *et al.* 2024).

A glutaconyl-CoA decarboxylase subunit with predicted involvement in butyrate production from amino acids and/or α-ketoglutarate was also identified by anvi’o in almost all *Lachnospiraceae* (33/36, versus 25/36 for Kofamscan and MicrobeAnnotator). Further analysis revealed that K23351 was always found as part of a conserved gene neighborhood including other subunits of this complex (Supplementary Fig. S11). The sequences found by anvi’o were also closely related to those found by Kofamscan and those included in KEGG GENES (Supplementary Fig. S12). Because methylmalonyl-CoA decarboxylase subunit sequences are also included in the KOfam profile for K23351, it is also possible that this protein has a role in synthesizing propionate, another short chain fatty acid that is a common gut microbial product. Experimental confirmation would be required to assign the function definitively, given the similarity between these protein families (Supplementary Fig. S12). However, these analyses do demonstrate that the extra anvi’o annotations for K23351 in the *Lachnospiraceae* have similar sequences and identical genomic context to those found by Kofamscan.

## 4 Conclusions

While many microbial genes will require sophisticated computational analyses of gene sequence (Vanni *et al.* 2022) and protein structure (Jumper *et al.* 2021) or labor-intensive experimental efforts (Hess *et al.* 2009) to determine their function, our results suggest that hundreds of genes in bacterial genomes may be overlooked by standard functional annotation approaches, with small tweaks leading to substantially higher recall. In particular, moderate and adaptive relaxation of bit score thresholds for pHMMs, as implemented in anvi’o, appears to improve annotation rates without large sacrifices in quality. Improved recovery of gene families with few existing representative sequences (here, nt-KOs) also allows us to identify more metabolic pathways with higher completeness. Reevaluating existing annotations using this heuristic may be beneficial, especially for organisms that are not well represented in culture collections.

## Supplementary Material

vbaf039_Supplementary_Data

## Data Availability

Scripts and workflow are available at https://github.com/pbradleylab/2023-anvio-comparison under the MIT license. Anvi’o is available at https://anvio.org under the GNU GPL license. Genomes are available through the NCBI GenBank database (Sayers *et al.* 2024); accessions are provided in Supplementary Table S1. High-resolution versions of all figures are available through Figshare under the CC-BY 4.0 license at https://doi.org/10.6084/m9.figshare.28331099.

## References

[vbaf039-B1] Altschul SF , Gish W, Miller W et al Basic local alignment search tool. J Mol Biol 1990;215:403–10. 2231712 10.1016/S0022-2836(05)80360-2

[vbaf039-B2] Aramaki T , Blanc-Mathieu R, Endo H et al KofamKOALA: KEGG ortholog assignment based on profile HMM and adaptive score threshold. Bioinformatics 2020;36:2251–2. 31742321 10.1093/bioinformatics/btz859 PMC7141845

[vbaf039-B3] Barrett T , Dowle M, Srinivasan A et al *data.table: extension of data.frame*. 2024. https://CRAN.R-project.org/package=data.table (7 May 2024, date last accessed).

[vbaf039-B4] Blin K. ncbi-genome-download. 2023. 10.5281/zenodo.8192486

[vbaf039-B5] Boeckmann B , Bairoch A, Apweiler R et al The SWISS-PROT protein knowledgebase and its supplement TrEMBL in 2003. Nucleic Acids Res 2003;31:365–70. 12520024 10.1093/nar/gkg095 PMC165542

[vbaf039-B6] Brämer CO , Silva LF, Gomez JGC et al Identification of the 2-methylcitrate pathway involved in the catabolism of propionate in the polyhydroxyalkanoate-producing strain *Burkholderia sacchari* IPT101(T) and analysis of a mutant accumulating a copolyester with higher 3-hydroxyvalerate content. Appl Environ Microbiol 2002;68:271–9. 11772636 10.1128/AEM.68.1.271-279.2002 PMC126583

[vbaf039-B7] Buchfink B , Reuter K, Drost H-G. Sensitive protein alignments at tree-of-life scale using DIAMOND. Nat Methods 2021;18:366–8. 33828273 10.1038/s41592-021-01101-x PMC8026399

[vbaf039-B8] Campitelli E. ggnewscale: Multiple Fill and Colour Scales in “ggplot2”. 2024. https://CRAN.R-project.org/package=ggnewscale

[vbaf039-B9] Cantalapiedra CP , Hernández-Plaza A, Letunic I et al eggNOG-mapper v2: functional annotation, orthology assignments, and domain prediction at the metagenomic scale. Mol Biol Evol 2021;38:5825–9. 34597405 10.1093/molbev/msab293 PMC8662613

[vbaf039-B10] Carroll J , Schep A, Sidi J. *ggeasy: Easy Access to “ggplot2” Commands*. 2024. https://CRAN.R-project.org/package=ggeasy

[vbaf039-B11] Castanheira M , Toleman MA, Jones RN et al Molecular characterization of a beta-lactamase gene, blaGIM-1, encoding a new subclass of metallo-beta-lactamase. Antimicrob Agents Chemother 2004;48:4654–61. 15561840 10.1128/AAC.48.12.4654-4661.2004 PMC529189

[vbaf039-B12] Cheng J , Liu W-Q, Zhu X et al Functional diversity of HemN-like proteins. ACS Bio Med Chem Au 2022;2:109–19. 10.1021/acsbiomedchemau.1c00058 PMC10114718 37101745

[vbaf039-B13] Dewi DAPR , Thomas T, Ahmad Mokhtar AM et al Carbapenem resistance among marine bacteria—an emerging threat to the global health sector. Microorganisms 2021;9:2147. 34683467 10.3390/microorganisms9102147 PMC8537846

[vbaf039-B14] Eddy SR. Accelerated profile HMM searches. PLoS Comput Biol 2011;7:e1002195. 22039361 10.1371/journal.pcbi.1002195 PMC3197634

[vbaf039-B15] Edgar RC. MUSCLE: multiple sequence alignment with high accuracy and high throughput. Nucleic Acids Res 2004;32:1792–7. 15034147 10.1093/nar/gkh340 PMC390337

[vbaf039-B16] Eren AM , Kiefl E, Shaiber A et al Community-led, integrated, reproducible multi-omics with anvi’o. Nat Microbiol 2021;6:3–6. 33349678 10.1038/s41564-020-00834-3 PMC8116326

[vbaf039-B17] Galperin MY , Koonin EV. Who’s your neighbor? New computational approaches for functional genomics. Nat Biotechnol 2000;18:609–13. 10835597 10.1038/76443

[vbaf039-B18] Garnier S et al 2024. viridis(Lite) - Colorblind-Friendly Color Maps for R. https://sjmgarnier.github.io/viridis/ (7 May 2024, date last accessed).

[vbaf039-B19] Gerike U , Hough DW, Russell NJ et al Citrate synthase and 2-methylcitrate synthase: structural, functional and evolutionary relationships. Microbiology (Reading) 1998;144:929–35. 9579066 10.1099/00221287-144-4-929

[vbaf039-B20] Gu Z. Complex heatmap visualization. iMeta 2022;1:e43. https://onlinelibrary.wiley.com/doi/10.1002/imt2.43 38868715 10.1002/imt2.43 PMC10989952

[vbaf039-B21] Gu Z , Eils R, Schlesner M. Complex heatmaps reveal patterns and correlations in multidimensional genomic data. Bioinformatics 2016;32:2847–9. 27207943 10.1093/bioinformatics/btw313

[vbaf039-B22] Hess DC , Myers CL, Huttenhower C et al Computationally driven, quantitative experiments discover genes required for mitochondrial biogenesis S. K. Kim, ed. PLoS Genet 2009;5:e1000407. 19300474 10.1371/journal.pgen.1000407 PMC2648979

[vbaf039-B23] Huerta-Cepas J , Szklarczyk D, Heller D et al eggNOG 5.0: a hierarchical, functionally and phylogenetically annotated orthology resource based on 5090 organisms and 2502 viruses. Nucleic Acids Res 2019;47:D309–14. 30418610 10.1093/nar/gky1085 PMC6324079

[vbaf039-B24] Hyatt D , Chen G-L, Locascio PF et al Prodigal: prokaryotic gene recognition and translation initiation site identification. BMC Bioinformatics 2010;11:119. 20211023 10.1186/1471-2105-11-119 PMC2848648

[vbaf039-B25] James P , Isupov MN, De Rose SA et al A “split-gene” transketolase from the hyper-thermophilic bacterium *Carboxydothermus hydrogenoformans*: structure and biochemical characterization. Front Microbiol 2020;11:592353. 33193259 10.3389/fmicb.2020.592353 PMC7661550

[vbaf039-B26] Jumper J , Evans R, Pritzel A et al Highly accurate protein structure prediction with AlphaFold. Nature 2021;596:583–9. 34265844 10.1038/s41586-021-03819-2 PMC8371605

[vbaf039-B27] Kanehisa M , Sato Y, Kawashima M et al KEGG as a reference resource for gene and protein annotation. Nucleic Acids Res 2016;44:D457–62. 26476454 10.1093/nar/gkv1070 PMC4702792

[vbaf039-B28] Koonin EV , Wolf YI. Genomics of bacteria and archaea: the emerging dynamic view of the prokaryotic world. Nucleic Acids Res 2008;36:6688–719. 18948295 10.1093/nar/gkn668 PMC2588523

[vbaf039-B29] Li F , Hagemeier CH, Seedorf H et al Re-citrate synthase from *Clostridium kluyveri* is phylogenetically related to homocitrate synthase and isopropylmalate synthase rather than to Si-citrate synthase. J Bacteriol 2007;189:4299–304. 17400742 10.1128/JB.00198-07 PMC1913417

[vbaf039-B30] Lin X , Hu T, Wu Z et al Isolation of potentially novel species expands the genomic and functional diversity of *Lachnospiraceae*. iMeta 2024;3:e174. https://onlinelibrary.wiley.com/doi/10.1002/imt2.174 38882499 10.1002/imt2.174 PMC11170972

[vbaf039-B31] Louis P , Flint HJ. Formation of propionate and butyrate by the human colonic microbiota. Environ Microbiol 2017;19:29–41. 27928878 10.1111/1462-2920.13589

[vbaf039-B32] Lu S , Wang J, Chitsaz F et al CDD/SPARCLE: the conserved domain database in 2020. Nucleic Acids Res 2020;48:D265–8. 31777944 10.1093/nar/gkz991 PMC6943070

[vbaf039-B33] Marchler-Bauer A , Panchenko AR, Shoemaker BA et al CDD: a database of conserved domain alignments with links to domain three-dimensional structure. Nucleic Acids Res 2002;30:281–3. 11752315 10.1093/nar/30.1.281 PMC99109

[vbaf039-B34] McKee RW , Aleksanyan N, Garrett EM et al Type IV Pili promote *Clostridium difficile* adherence and persistence in a mouse model of infection. Infect Immun 2018;86:e00943–17. 29483294 10.1128/IAI.00943-17 PMC5913833

[vbaf039-B35] Mölder F , Jablonski KP, Letcher B et al Sustainable data analysis with Snakemake. F1000Res 2021;10:33. 34035898 10.12688/f1000research.29032.1 PMC8114187

[vbaf039-B36] O’Leary NA et al Reference sequence (RefSeq) database at NCBI: current status, taxonomic expansion, and functional annotation. Nucleic Acids Res 2016;44:D733–45. 26553804 10.1093/nar/gkv1189 PMC4702849

[vbaf039-B37] Ooms J. The jsonlite Package: a practical and consistent mapping between JSON data and R objects. arXiv, arXiv:1403.2805, 2014, preprint: not peer reviewed.

[vbaf039-B38] Overbeek R , Fonstein M, D'Souza M et al The use of gene clusters to infer functional coupling. Proc Natl Acad Sci USA 1999;96:2896–901. 10077608 10.1073/pnas.96.6.2896 PMC15866

[vbaf039-B39] Pagès H , Aboyoun P, Gentleman R et al Biostrings: efficient manipulation of biological strings. 2024. https://bioconductor.org/packages/Biostrings

[vbaf039-B40] Paradis E , Schliep K. ape 5.0: an environment for modern phylogenetics and evolutionary analyses in R. Bioinformatics 2019;35:526–8. 10.1093/bioinformatics/bty633 30016406

[vbaf039-B41] Parks DH , Chuvochina M, Chaumeil P-A et al A complete domain-to-species taxonomy for bacteria and archaea. Nat Biotechnol 2020;38:1079–86. 32341564 10.1038/s41587-020-0501-8

[vbaf039-B42] Parks DH , Chuvochina M, Waite DW et al A standardized bacterial taxonomy based on genome phylogeny substantially revises the tree of life. Nat Biotechnol 2018;36:996–1004. 30148503 10.1038/nbt.4229

[vbaf039-B43] Parks DH , Chuvochina M, Rinke C et al GTDB: an ongoing census of bacterial and archaeal diversity through a phylogenetically consistent, rank normalized and complete genome-based taxonomy. Nucleic Acids Res 2022;50:D785–94. 34520557 10.1093/nar/gkab776 PMC8728215

[vbaf039-B44] Pedersen TL. *patchwork: The Composer of Plots*. 2024. https://github.com/thomasp85/patchwork

[vbaf039-B45] Price MN , Dehal PS, Arkin AP. FastTree 2—approximately maximum-likelihood trees for large alignments. PLoS One 2010;5:e9490. 20224823 10.1371/journal.pone.0009490 PMC2835736

[vbaf039-B46] R Core Team. R: A Language and Environment for Statistical Computing. Vienna, Austria: R Foundation for Statistical Computing. 2024. https://www.R-project.org/

[vbaf039-B47] Revell LJ. phytools 2.0: an updated R ecosystem for phylogenetic comparative methods (and other things). PeerJ 2024;12:e16505. 10.7717/peerj.16505 38192598 PMC10773453

[vbaf039-B48] Richardson N , Cook I, Crane N et al *arrow: Integration to “Apache” “Arrow”*. 2024. https://CRAN.R-project.org/package=arrow

[vbaf039-B49] Rinke C , Chuvochina M, Mussig AJ et al A standardized archaeal taxonomy for the genome taxonomy database. Nat Microbiol 2021;6:946–59. 34155373 10.1038/s41564-021-00918-8

[vbaf039-B50] Ruiz-Perez CA , Conrad RE, Konstantinidis KT. MicrobeAnnotator: a user-friendly, comprehensive functional annotation pipeline for microbial genomes. BMC Bioinformatics 2021;22:11. 33407081 10.1186/s12859-020-03940-5 PMC7789693

[vbaf039-B51] Sayers EW , Cavanaugh M, Clark K et al GenBank 2024 update. Nucleic Acids Res 2024;52:D134–7. 37889039 10.1093/nar/gkad903 PMC10767886

[vbaf039-B52] Sheffield NC , Stolarczyk M, Reuter VP et al Linking big biomedical datasets to modular analysis with portable encapsulated projects. Gigascience 2021;10:giab077. 10.1093/gigascience/giab077 PMC8673555 34890448

[vbaf039-B53] Shimoyama Y. pyMSAviz: MSA visualization python package for sequence analysis. 2022. https://github.com/moshi4/pyMSAviz (8 November 2024, date last accessed).

[vbaf039-B54] Sievers F , Wilm A, Dineen D et al Fast, scalable generation of high-quality protein multiple sequence alignments using clustal omega. Mol Syst Biol 2011;7:539. 21988835 10.1038/msb.2011.75 PMC3261699

[vbaf039-B55] Sievers F , Higgins DG. Clustal Omega for making accurate alignments of many protein sequences. Protein Sci 2018;27:135–45. 28884485 10.1002/pro.3290 PMC5734385

[vbaf039-B56] Sievert C. Interactive Web-Based Data Visualization with R, Plotly, and Shiny. Chapman and Hall/CRC. 2019.

[vbaf039-B57] Storey JD , Bass AJ, Dabney A et al *qvalue: Q-value estimation for false discovery rate control*. 2015. http://github.com/jdstorey/qvalue

[vbaf039-B58] UniProt Consortium. UniProt: the universal protein knowledgebase in 2023. Nucleic Acids Res 2023;51:D523–31. 36408920 10.1093/nar/gkac1052 PMC9825514

[vbaf039-B59] Vanni C , Schechter MS, Acinas SG et al Unifying the known and unknown microbial coding sequence space. eLife 2022;11:e67667. 10.7554/eLife.67667 PMC9132574 35356891

[vbaf039-B60] Veseli I , Chen YT, Schechter MS et al Microbes with higher metabolic independence are enriched in human gut microbiomes under stress. eLife 2023;12:RP89862. 10.7554/eLife.89862 PMC12084026 40377187

[vbaf039-B61] Vital M , Howe AC, Tiedje JM. Revealing the bacterial butyrate synthesis pathways by analyzing (meta)genomic data. mBio 2014;5:e00889. 24757212 10.1128/mBio.00889-14 PMC3994512

[vbaf039-B62] Waller PR , Sauer RT. Characterization of degQ and degS, *Escherichia coli* genes encoding homologs of the DegP protease. J Bacteriol 1996;178:1146–53. 8576051 10.1128/jb.178.4.1146-1153.1996 PMC177778

[vbaf039-B63] Wang J , Chitsaz F, Derbyshire MK et al The conserved domain database in 2023. Nucleic Acids Res 2023;51:D384–8. 36477806 10.1093/nar/gkac1096 PMC9825596

[vbaf039-B64] Wickham H , Hester J, Chang W et al *devtools: Tools to Make Developing R Packages Easier*. 2022. https://CRAN.R-project.org/package=devtools

[vbaf039-B65] Wickham H. ggplot2: Elegant Graphics for Data Analysis. New York: Springer-Verlag. 2016.

[vbaf039-B66] Wickham H. The Split-Apply-Combine strategy for data analysis. J Stat Soft 2011;40:1–29.

[vbaf039-B67] Wickham H , Averick M, Bryan J et al Welcome to the tidyverse. JOSS 2019;4:1686.

[vbaf039-B68] Wilkins D. *gggenes: Draw Gene Arrow Maps in “ggplot2”*. 2023. https://CRAN.R-project.org/package=gggenes

[vbaf039-B69] Xu S , Dai Z, Guo P et al ggtreeExtra: compact visualization of richly annotated phylogenetic data. Mol Biol Evol 2021;38:4039–42. https://academic.oup.com/mbe/advance-article/doi/10.1093/molbev/msab166/6294410 34097064 10.1093/molbev/msab166 PMC8382893

[vbaf039-B70] Yang M , Derbyshire MK, Yamashita RA et al NCBI’s conserved domain database and tools for protein domain analysis. Curr Protoc Bioinf 2020;69:e90. 10.1002/cpbi.90 PMC7378889 31851420

[vbaf039-B71] Yu G , Smith DK, Zhu H et al ggtree: an R package for visualization and annotation of phylogenetic trees with their covariates and other associated data. Methods Ecol Evol 2017;8:28–36.

[vbaf039-B72] Zhou L , Feng T, Xu S et al ggmsa: a visual exploration tool for multiple sequence alignment and associated data. Brief Bioinform 2022;23:06. https://academic.oup.com/bib/article-abstract/23/4/bbac222/6603927 10.1093/bib/bbac222 35671504

