# Supplementary material for: Adaptive adjustment of profile HMM significance thresholds improves functional and metabolic insights into microbial genomes
Source: Bioinform Adv. 2025 Mar 21;5(1):vbaf039. doi: 10.1093/bioadv/vbaf039 (PMC11964587; doi:10.1093/bioadv/vbaf039)
Supplement: vbaf039_Supplementary_Data [file vbaf039_supplementary_data.zip › AdaptiveAdjustment-Revision-SuppInfo.pdf]

Supplementary Information

Detailed methodology of benchmarked software

MicrobeAnnotator [2], Kofamscan [1], and the `anvi-run-kegg-kofams` command in anvi'o [3] are programs that annotate KOs by querying the KOfam database via the HMMER software [72]. Most pHMMs in the KOfam database are associated with a predefined bit score threshold that these tools use to differentiate between strong and weak matches to the model. That is, when a query gene sequence has some homology to a KOfam model, the sequence is considered homologous enough for annotation when the match's bit score is at or above the model's predefined bit score threshold.

The primary difference between these tools' annotation methods lies in their strategy for enabling the annotation of additional below-threshold to KEGG Orthologs. MicrobeAnnotator addresses this problem by the use of multiple reference databases beyond KEGG and enabling the conversion of annotations from these databases into KOs (via the `--refine` parameter). Kofamscan, on the other hand, implements an optional parameter for the relaxation of bit score thresholds (`--threshold-scale`) that applies globally (i.e., across all KOfam models), and additionally enables expectation value thresholding via the `--e-value` parameter. The anvi'o program `anvi-run-kegg-kofams` takes a more nuanced approach to relaxing bit score thresholds: it re-visits genes that are not annotated using the predefined bit score thresholds and assesses the set of HMM hits to these genes with bit scores above a given fraction of the KOfam model's original bit score threshold (default fraction is 0.5) and e-values below a given e-value threshold (by default, 1e-05). If all of these slightly-less homologous hits are from a unique KO model, then the gene is annotated with that KO (Supplementary Figure 1).

Some KOfam models (henceforth referred to as 'nt-KOs' to stand for 'no-threshold KOs') lack a bit score threshold for differentiating between strong and weak hits because they are built from too few sequences to use the threshold estimation workflow described in Aramaki et al. [1], and the three tools also differ from one another in how they handle annotations to these gene families while additionally providing an alternative to using a pairwise approach. MicrobeAnnotator annotates any gene with a match to one of these nt-KOs regardless of the match's similarity score. Depending on the requested output format, Kofamscan either does not annotate these nt-KOs at all or adds an asterisk beside the annotation in the output file to indicate its potentially dubious nature. Anvi'o by default does not use these KOfam models but allows users to include them in its annotation workflow via the `--include-stray-KOs` parameter, which relies upon a conservative bit score threshold computed within anvi'o for each of these models by taking the minimum bit score of matches between the model and the gene sequences used to create it. These approaches differ in their potential to introduce false positive annotations via the inclusion of poor matches to this subset of KOfam models without predefined bit score thresholds.
