# Supplementary material for: Adaptive adjustment of profile HMM significance thresholds improves functional and metabolic insights into microbial genomes
Source: Bioinform Adv. 2025 Mar 21;5(1):vbaf039. doi: 10.1093/bioadv/vbaf039 (PMC11964587; doi:10.1093/bioadv/vbaf039)
Supplement: vbaf039_Supplementary_Data [file vbaf039_supplementary_data.zip › AdaptiveAdjustment-SuppFigsTables.pdf]

Supplementary Figures

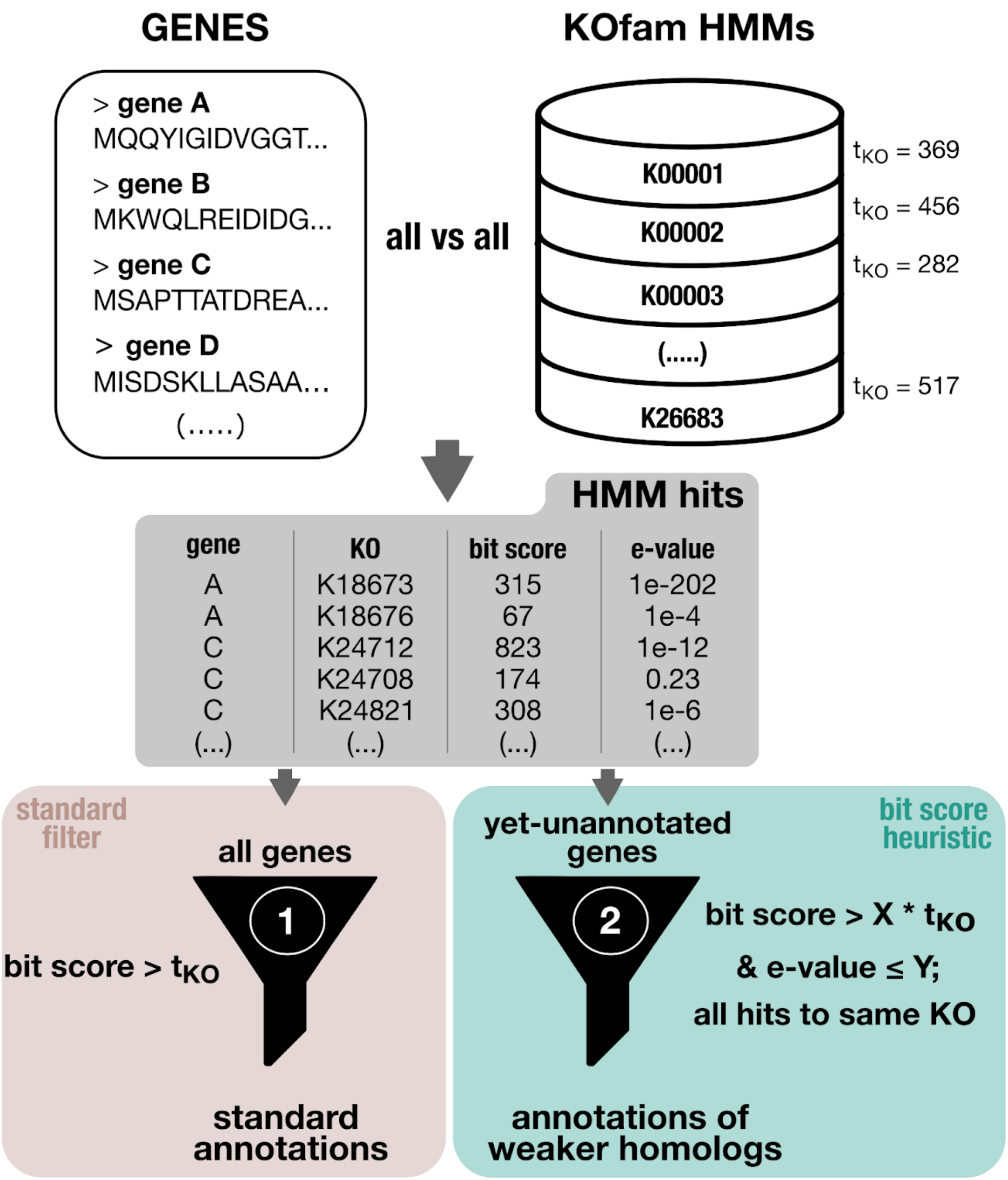

Supplementary Figure 1. The annotation workflow and bit score heuristic in anvi'o. In `anvi-run-kegg-kofams`, HMM hits from an all vs. all query of predicted gene sequences against the KOfam database are initially filtered using the KEGG-defined bit score threshold for each KO model, t_{KO}. If the bit score heuristic is turned on (the default behavior, in green), the program does a second pass through the hits to genes that have not yet been annotated, filtering them using a percentage (X) of the original bit score threshold and a maximum e-value cut-off (Y). If all of those filtered hits are to the same KO model for a given gene, the gene is annotated with the KO despite its slightly weaker homology to this family.

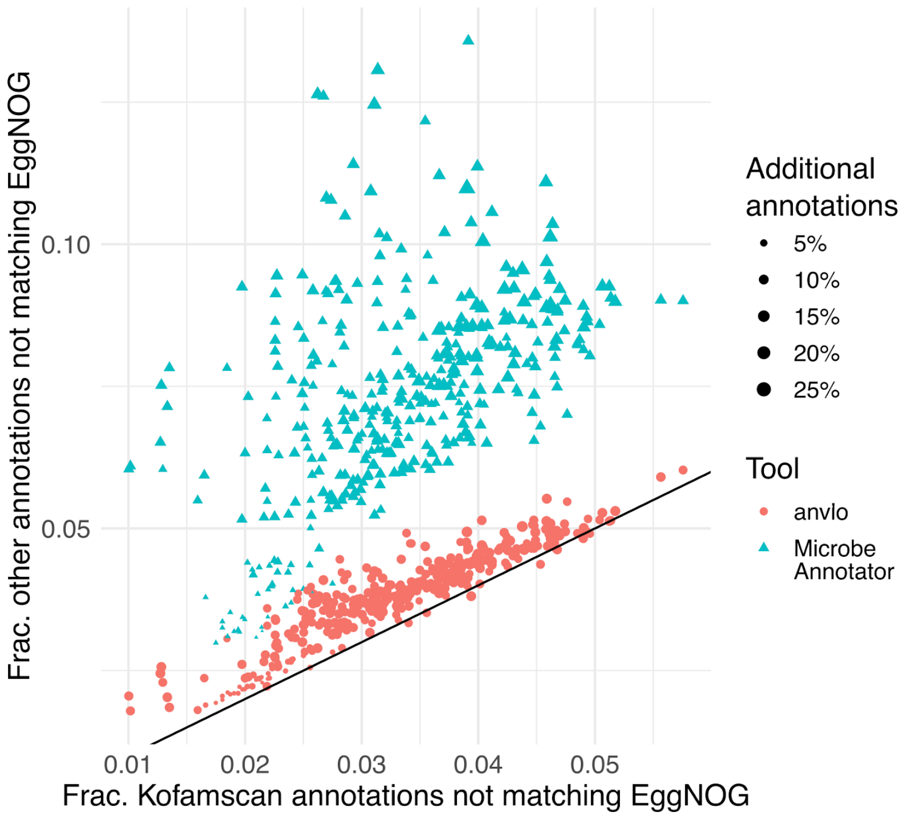

Supplementary Figure 2. Per-genome fraction of profile HMM KOfam annotations that disagreed with EggNOG-mapper. Each point is one genome. The x-axis gives the fraction of annotations that disagreed for Kofamscan, and the y-axis gives the same number for either anvi'o (red) or MicrobeAnnotator (teal). Point size corresponds to the total number of additional annotations over Kofamscan, and the equality line (i.e., the line connecting the same values on both axes) is marked in black.

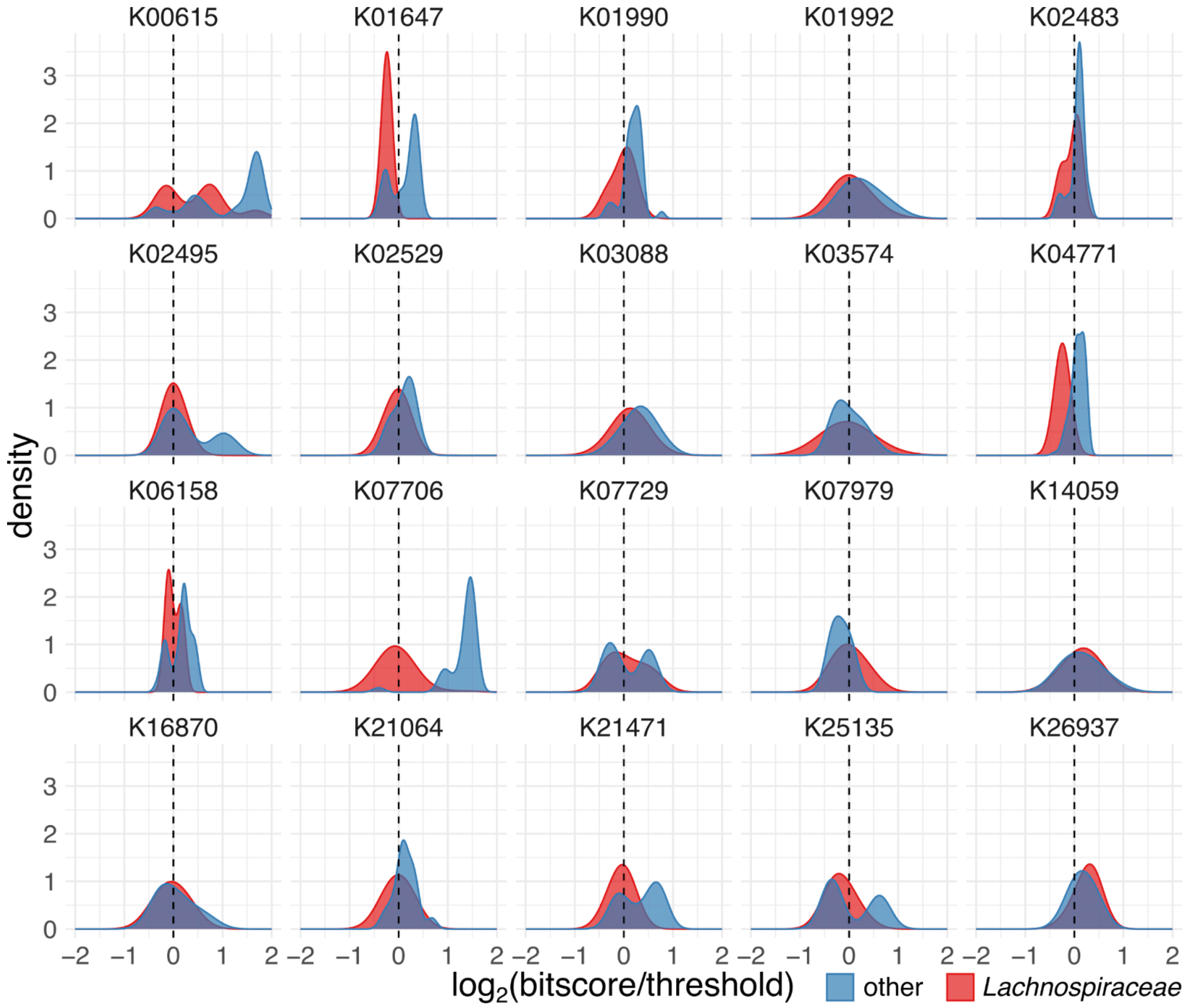

Supplementary Figure 3. Per-KOfam density plots of normalized bit scores (log_2-ratios to the Kofamscan threshold; the dashed vertical line at zero indicates this threshold) for annotations made using anvi'o. The 20 KOfams with the most additional anvi'o annotations in the *Lachnospiraceae* are plotted. Normalized bit score distributions are plotted separately for *Lachnospiraceae* annotations (red) and for annotations in all other taxa (blue).

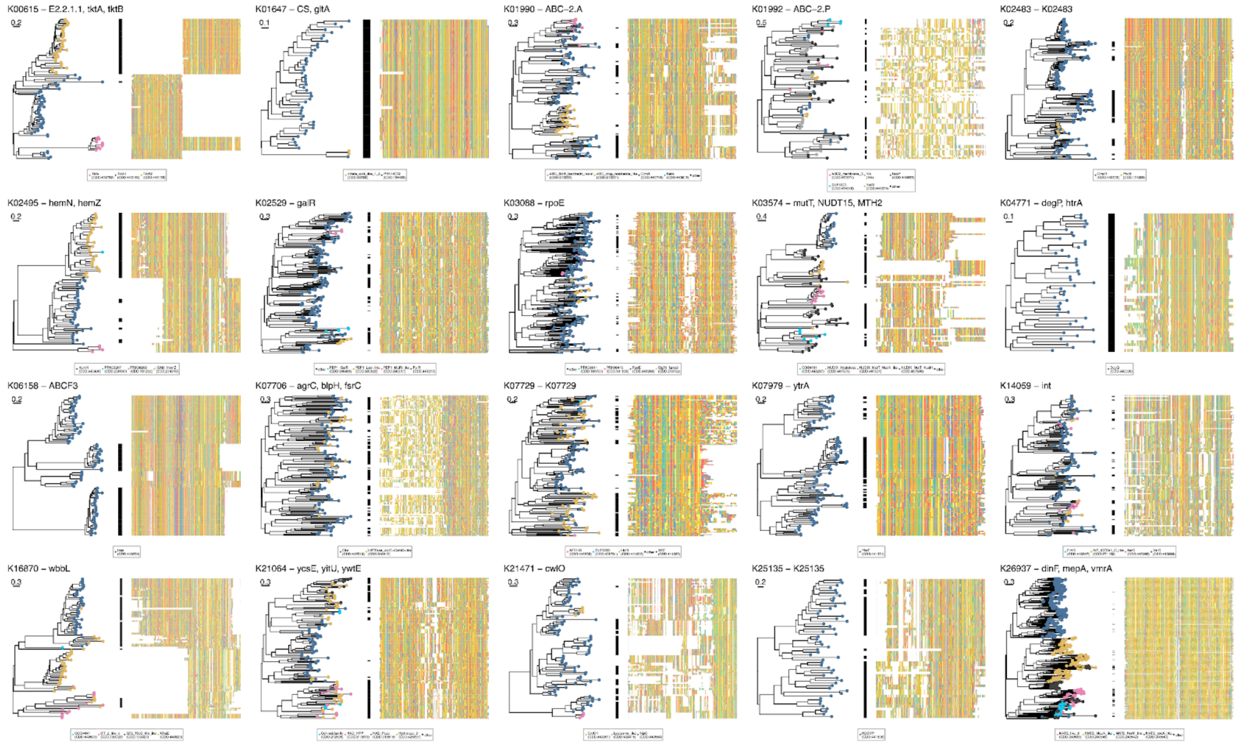

Supplementary Figure 4. Gene trees with scale bars (left panels) and multiple sequence alignments (right panels) for the KOfams in Supplementary Figure 3. Gene trees are colored by the best conserved domain (CD) family that they matched using RPS-BLAST.

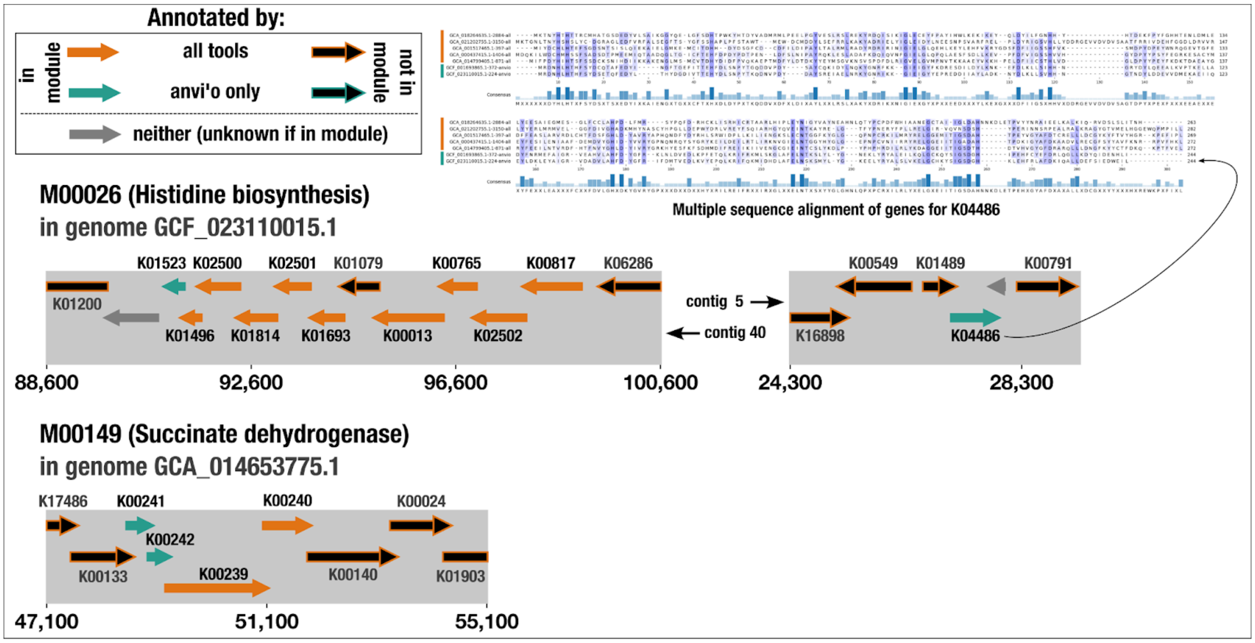

Supplementary Figure 5. Additional examples of modules in operon-like structures that are more complete using anvi'o annotations. The gene synteny of each module is shown within one genome, but similar organization was seen across multiple genomes. Genes are shown as arrows that indicate whether they are on the forward or reverse strand. Each gene is colored or outlined according to which tool(s), if any, were able to annotate it in the displayed genome. Full-color arrows indicate genes belonging to the example module, black arrows indicate genes that do not belong to the module, and gray arrows indicate genes without any functional annotation. Annotation counts for the KOs in these modules across all genomes can be found in Supplementary Table 3. A multiple sequence alignment of selected gene sequences for K04486, which is isolated from the gene neighborhood of module M00026 in genome GCF_023110015.1, is shown in the upper left corner. The colored bars next to the sequence labels indicate which tool(s) were able to annotate each sequence.

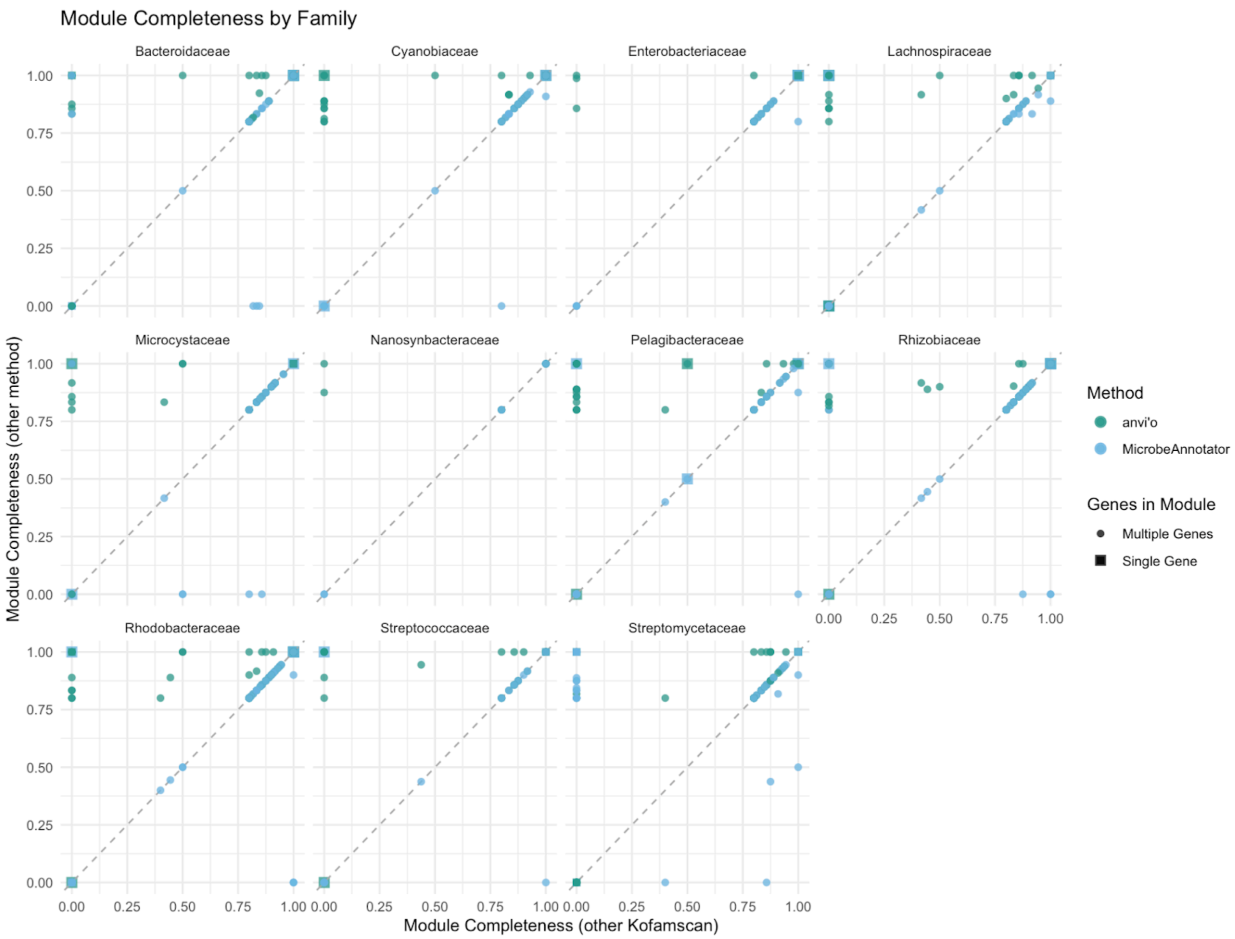

Supplementary Figure 6. Median module completeness of Kofamscan (x-axis) compared to anvi'o (y-axis, green) and MicrobeAnnotator (y-axis, blue). Each point is the median completeness score of one module over all genomes in a given bacterial family (facets) with point shape indicating the number of KOs in a module ($x \geq 2$ or $x = 1$).

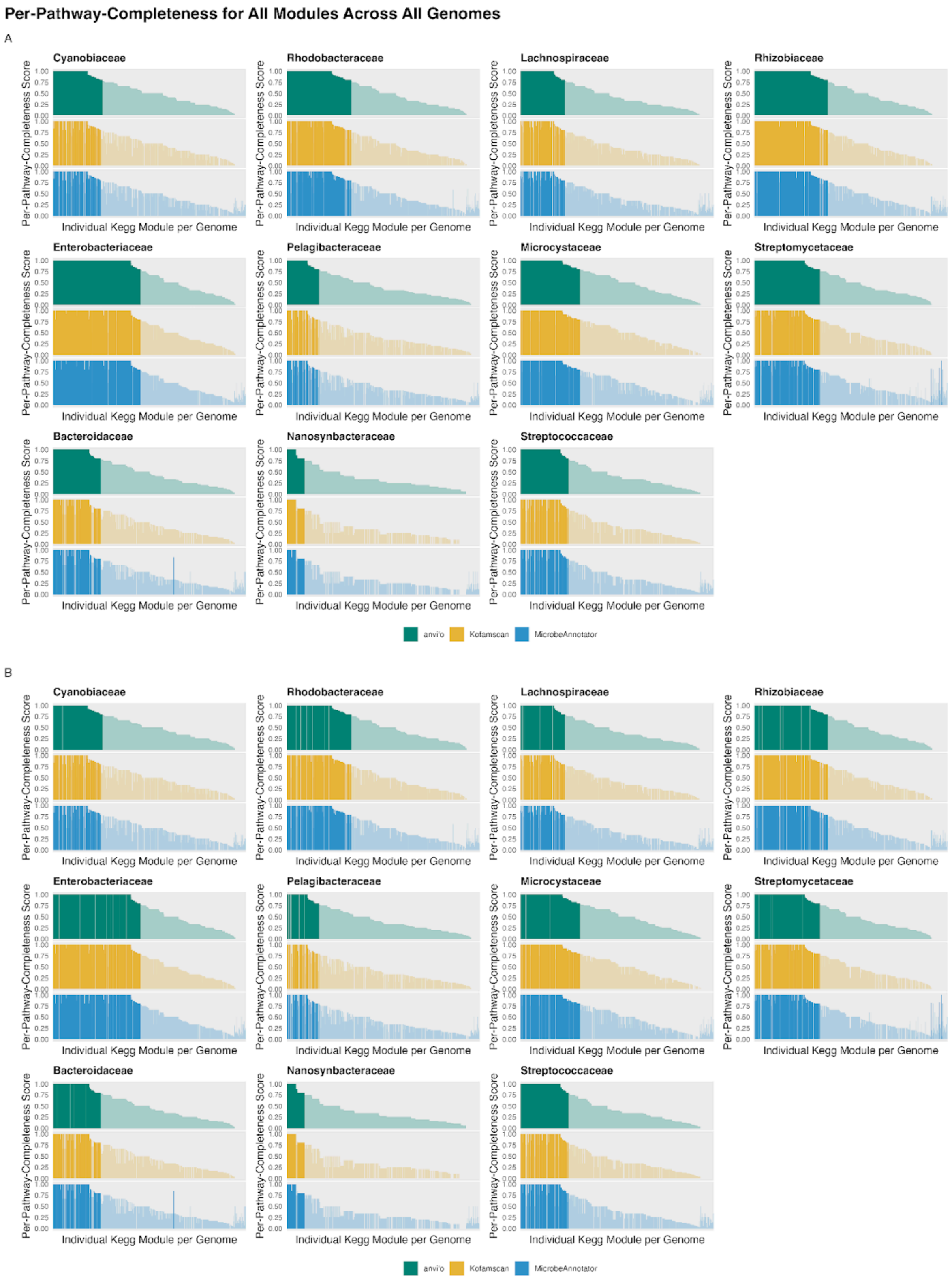

Supplementary Figure 7. The per-pathway completeness distribution across all modules, for each tool, in every species. **A**. Darker colors show modules at x ≥ 80% completeness. **B.** Darker colors show modules at x ≥ 80% completeness and with 2 or more KOs.

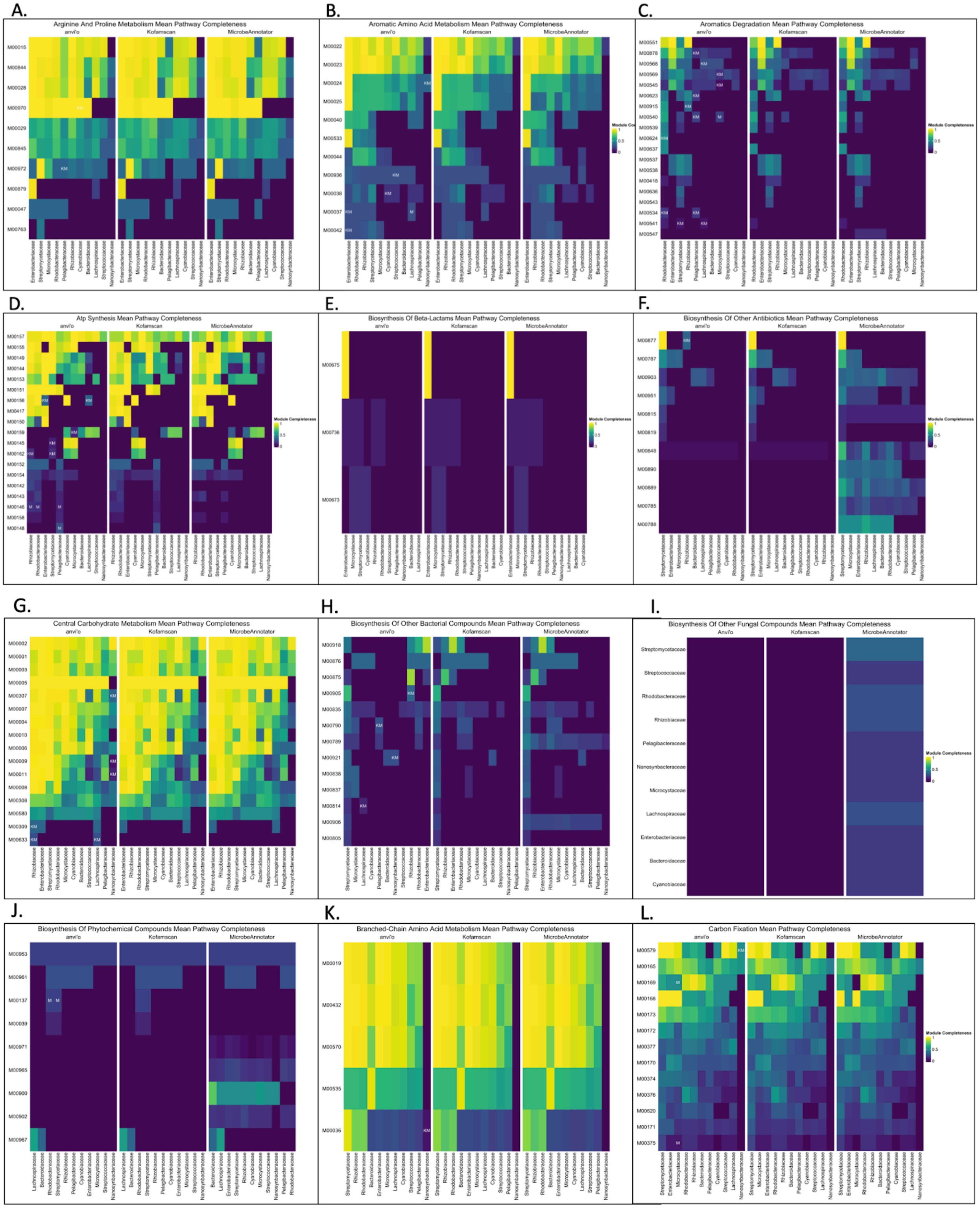
A.
Arginine And Proline Metabolism Mean Pathway Completeness
B.
Aromatic Amino Acid Metabolism Mean Pathway Completeness
C.
Aromatics Degradation Mean Pathway Completeness
D.
Atp Synthesis Mean Pathway Completeness
E.
Biosynthesis Of Beta-Lactams Mean Pathway Completeness
F.
Biosynthesis Of Other Antibiotics Mean Pathway Completeness
G.
Central Carbohydrate Metabolism Mean Pathway Completeness
H.
Biosynthesis Of Other Bacterial Compounds Mean Pathway Completeness
I.
Biosynthesis Of Other Fungal Compounds Mean Pathway Completeness
J.
Biosynthesis Of Phytochemical Compounds Mean Pathway Completeness
K.
Branched-Chain Amino Acid Metabolism Mean Pathway Completeness
L.
Carbon Fixation Mean Pathway Completeness
anvi'o
Kofamscan
MicrobeAnnotator
Module Completeness

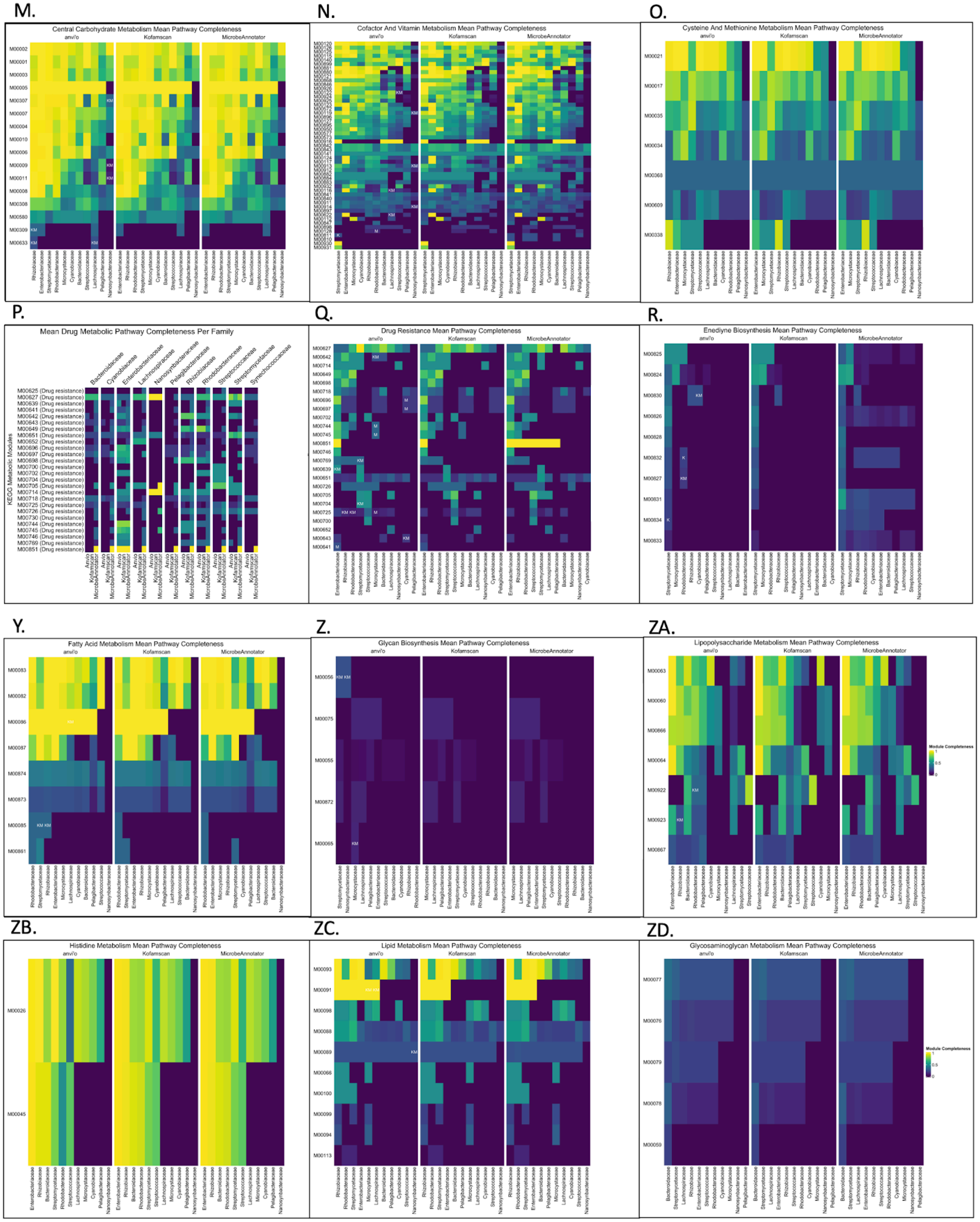
M.
Central Carbohydrate Metabolism Mean Pathway Completeness
N.
Cofactor And Vitamin Metabolism Mean Pathway Completeness
O.
Cysteine And Methionine Metabolism Mean Pathway Completeness
P.
Mean Drug Metabolic Pathway Completeness Per Family
KEGG Metabolic Modules
Q.
Drug Resistance Mean Pathway Completeness
R.
Enediyne Biosynthesis Mean Pathway Completeness
Y.
Fatty Acid Metabolism Mean Pathway Completeness
Z.
Glycan Biosynthesis Mean Pathway Completeness
ZA.
Lipopolysaccharide Metabolism Mean Pathway Completeness
Module Completeness
ZB.
Histidine Metabolism Mean Pathway Completeness
ZC.
Lipid Metabolism Mean Pathway Completeness
ZD.
Glycosaminoglycan Metabolism Mean Pathway Completeness
Module Completeness
anvi'o
Kofamscan
MicrobeAnnotator

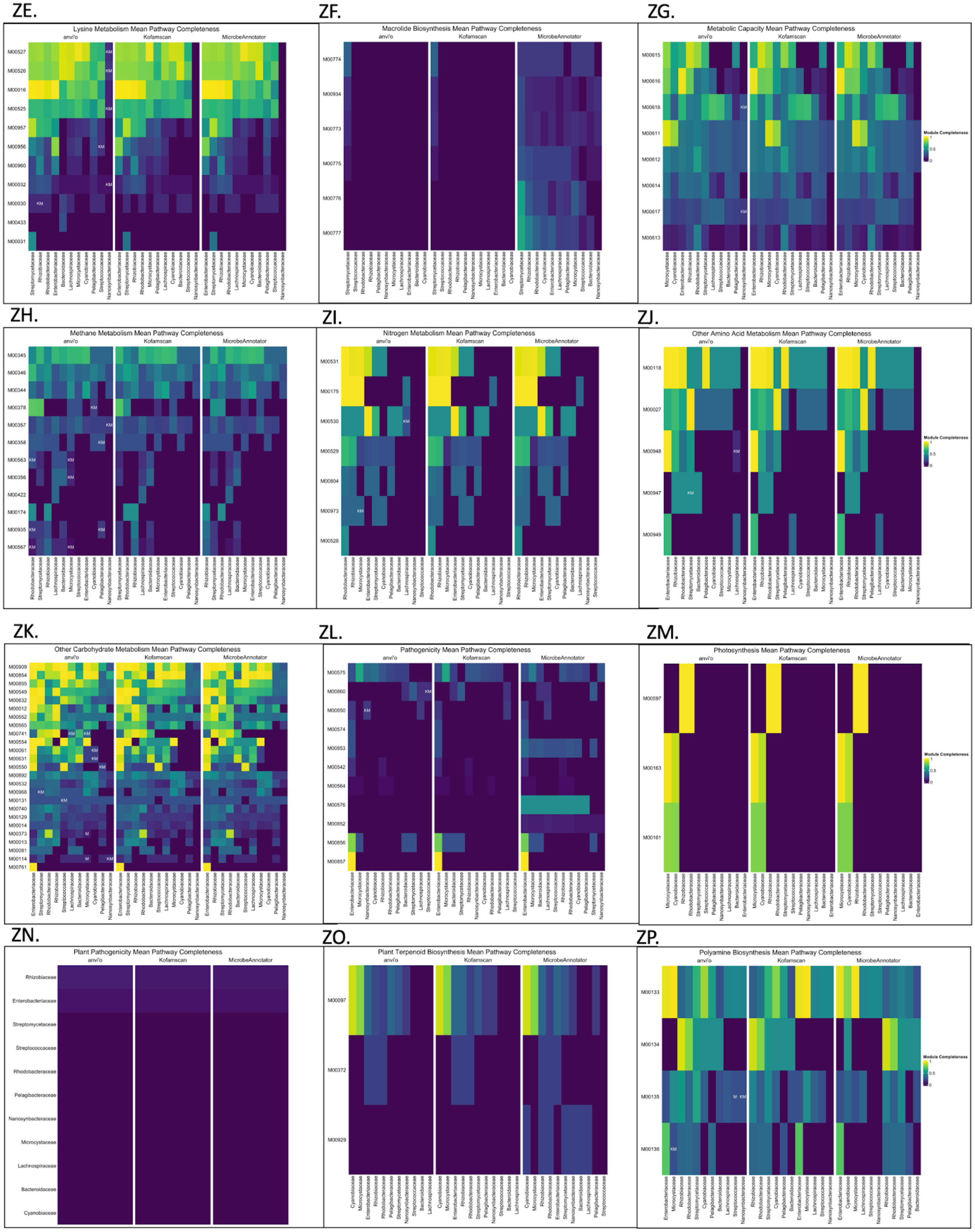
ZE.
Lysine Metabolism Mean Pathway Completeness
anvi'o
Kofamscan
MicrobeAnnotator
ZF.
Macrolide Biosynthesis Mean Pathway Completeness
ZG.
Metabolic Capacity Mean Pathway Completeness
Module Completeness
ZH.
Methane Metabolism Mean Pathway Completeness
ZI.
Nitrogen Metabolism Mean Pathway Completeness
ZJ.
Other Amino Acid Metabolism Mean Pathway Completeness
ZK.
Other Carbohydrate Metabolism Mean Pathway Completeness
ZL.
Pathogenicity Mean Pathway Completeness
ZM.
Photosynthesis Mean Pathway Completeness
ZN.
Plant Pathogenicity Mean Pathway Completeness
ZO.
Plant Terpenoid Biosynthesis Mean Pathway Completeness
ZP.
Polyamine Biosynthesis Mean Pathway Completeness

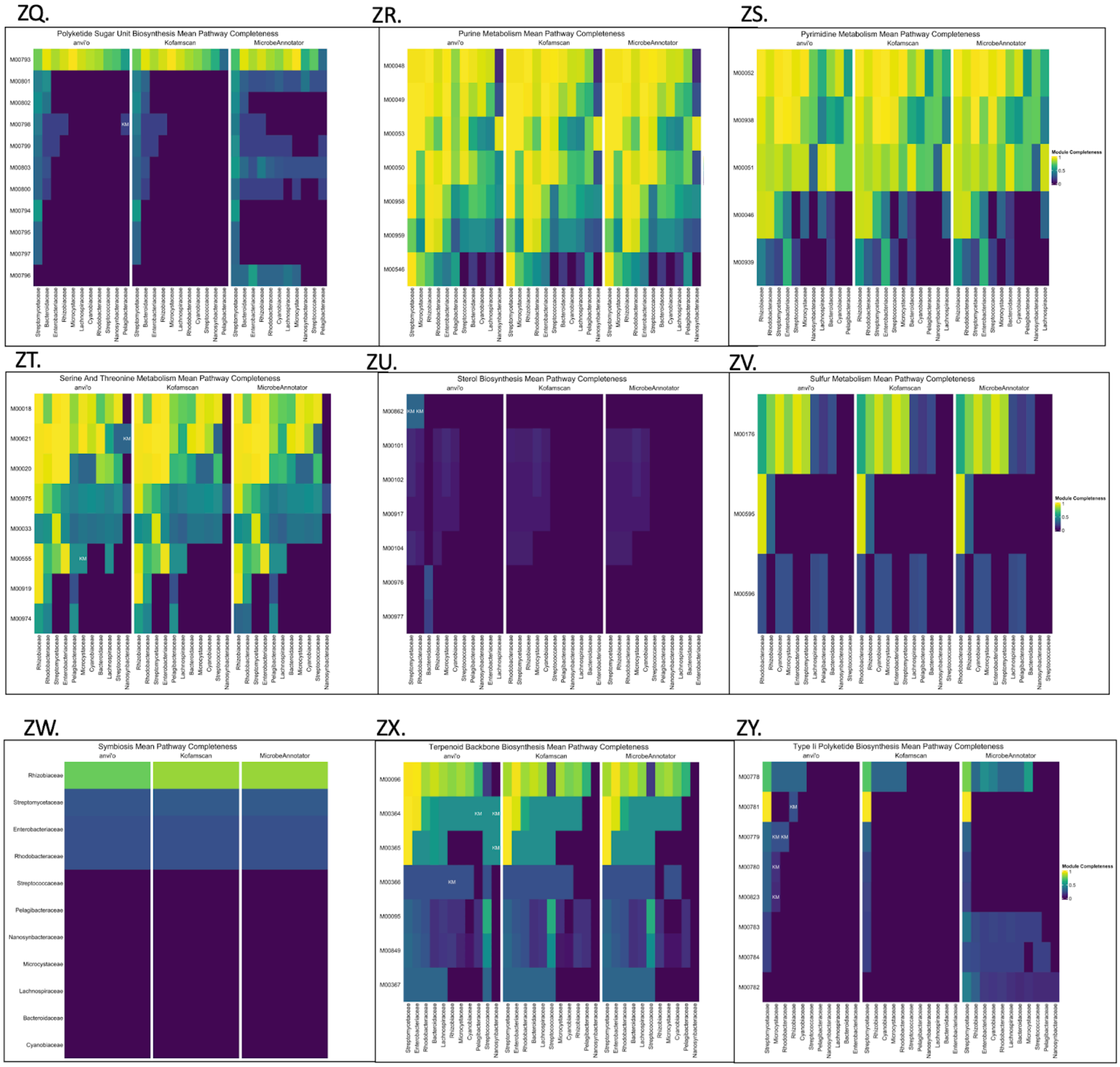

Supplementary Figure 8. The mean module completeness score in each microbial family for all KEGG modules found across the three methods is shown. Each module is grouped into one of 45 categories and the MicrobeAnnotator, Kofamscan, and anvi'o default results are compared. Modules predicted with >0% completeness by anvi'o are marked with an 'M' if they are not also found with MicrobeAnnotator, a 'K' if they are not found with Kofamscan, and 'KM' if they are absent (0% complete) in both of the other methods.

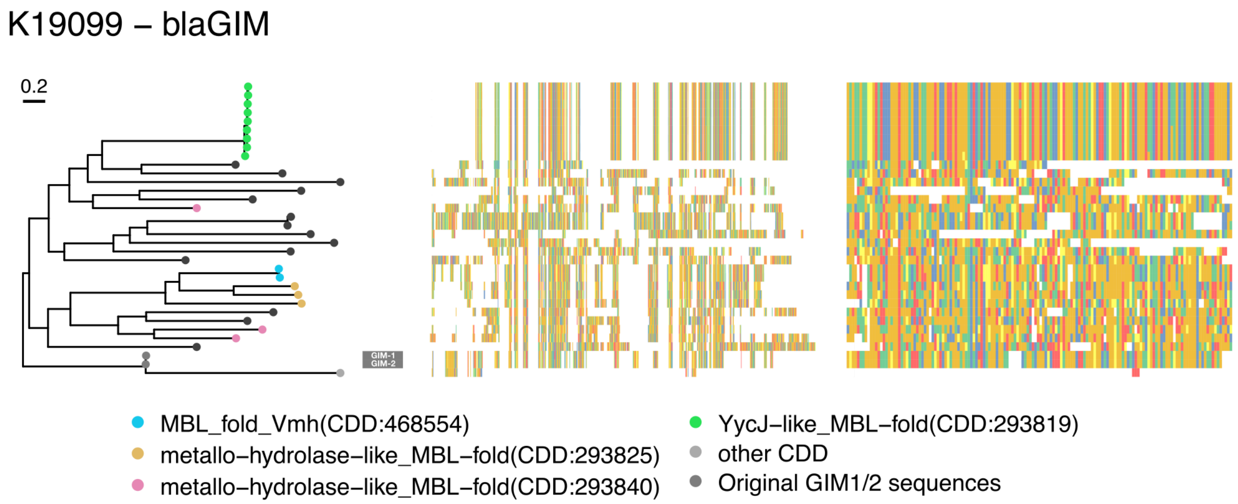

Supplementary Figure 9. Gene tree with scale bar and alignment of MicrobeAnnotator hits to the KO K19099 (metallo-beta-lactamase class B GIM). The only two reference sequences (GIM-1, GIM-2) from the KEGG GENES database have been labeled in dark gray with white text. The middle alignment shows the full multiple sequence alignment for all sequences; in the alignment to the far right, only columns with 25% or fewer gaps have been retained.

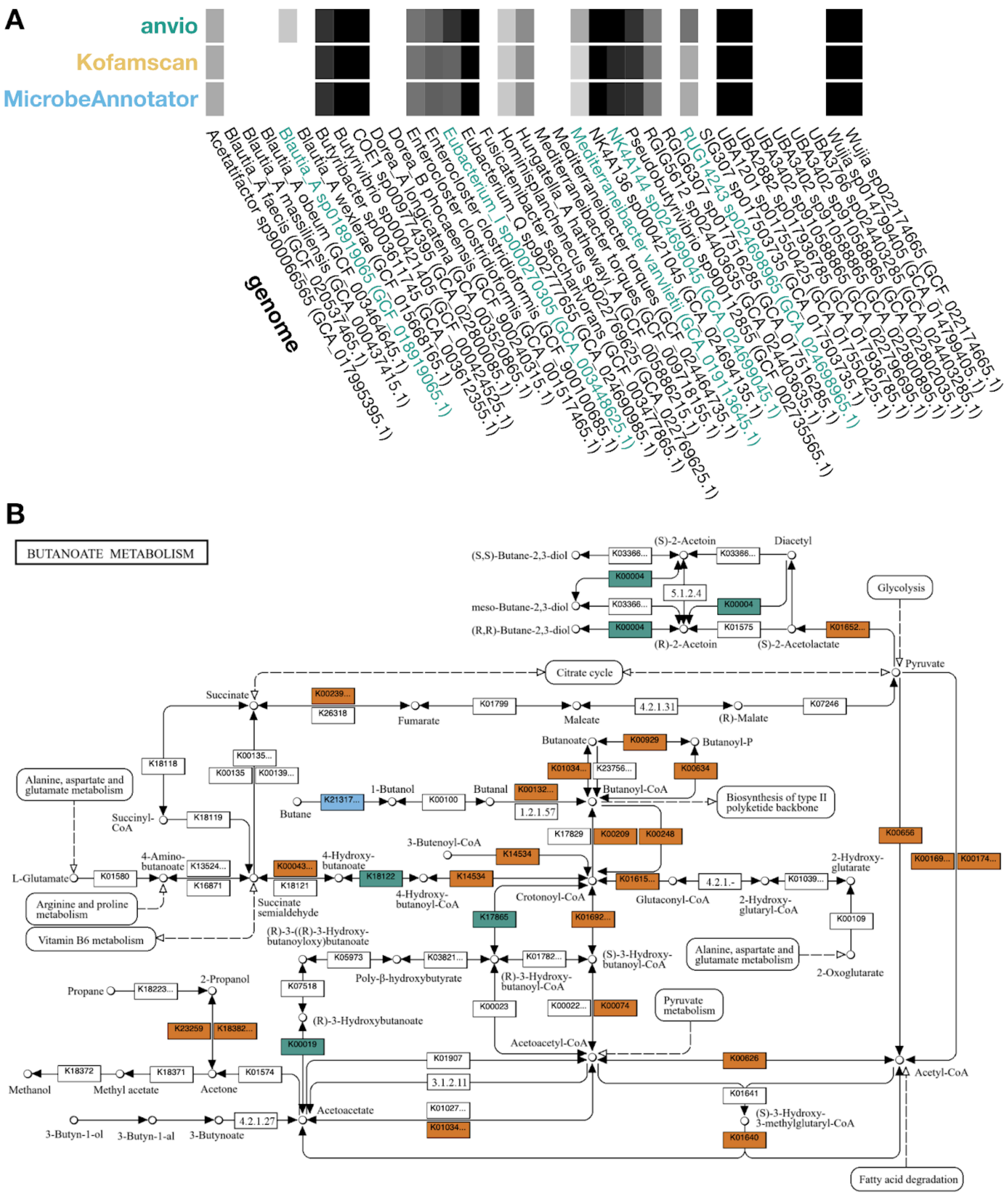

Supplementary Figure 10. The butyrate biosynthesis pathway as annotated in the 36 *Lachnospiraceae* genomes in our test dataset. Each tool was run with default parameters. **A.** Heatmap of pathwise completeness scores for the custom butyrate biosynthesis pathway module defined with substrate-specific enzymes whenever possible (the "SUBSPEC" module). Genome labels are colored green when anvi'o estimates higher completeness for the pathway than the other tools. **B.** The combined annotations from all 36 genomes mapped onto the KEGG Pathway Map for Butanoate Metabolism and colored according to which tool(s) could annotate

them. Enzymatic reactions are colored orange if anvi'o, MicrobeAnnotator, and KOfamscan all annotated the corresponding orthologs, green if anvi'o alone annotated orthologs, and blue if MicrobeAnnotator alone annotated orthologs. No other combinations of tools were observed in the ortholog annotations for this pathway map.

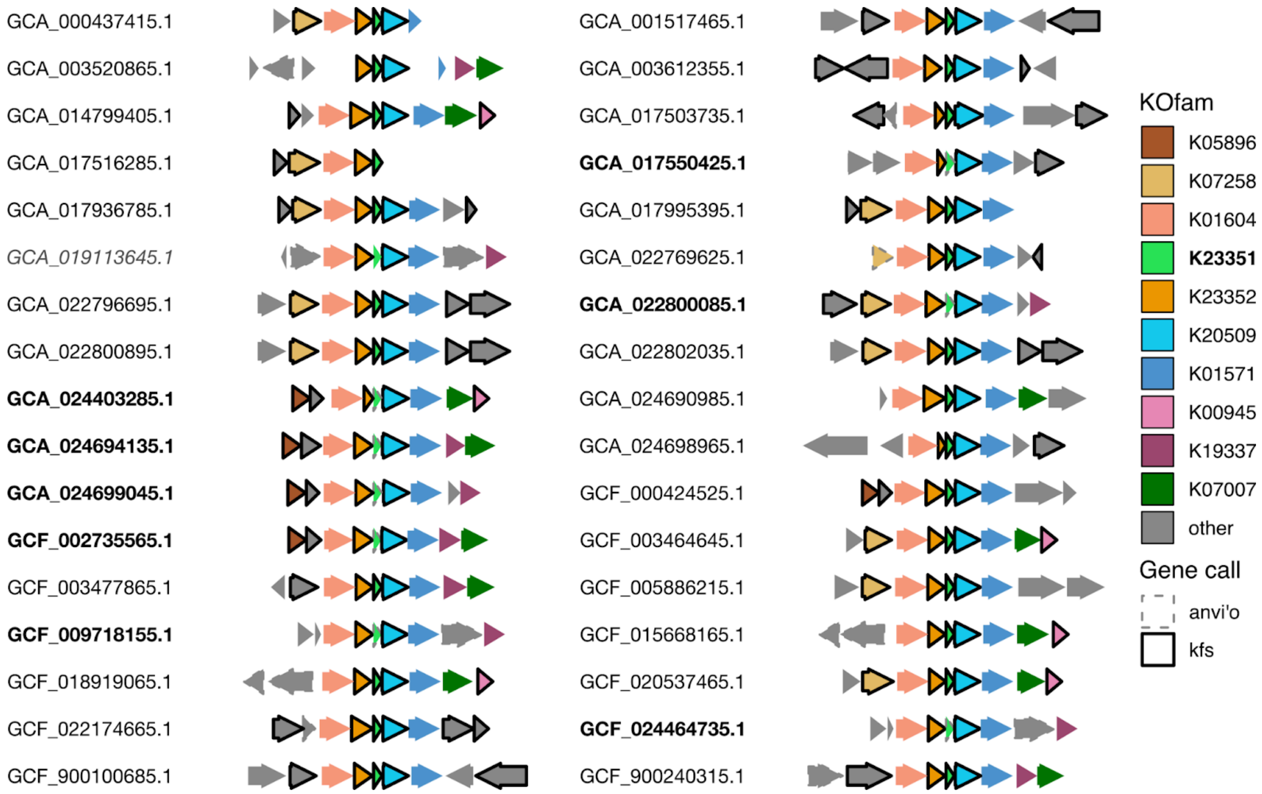

Supplementary Figure 11. *Lachnospiraceae* gene neighborhoods centered on glutaconyl-CoA/methylmalonyl-CoA decarboxylase, subunit gamma (K23351). Out of 36 tested *Lachnospiraceae* genomes, 34 encode proteins whose best KOfam match is K23351. Neighboring genes on the same contig (arrows), including up to four features in either direction, are colored by their best KOfam match. For visualization, the orientation of each region is flipped such that K23352 is always upstream of K23351. Matches found by Kofamscan and anvi'o are outlined in solid black, matches found only by anvi'o are outlined in dashed gray, and matches below both the Kofamscan and the anvi'o heuristic thresholds are not outlined. Eight genomes where only anvi'o found K23351 are shown in bold text; one genome where the best hit to K23351 was below both thresholds is shown in italics. The middle three genes in this neighborhood are K23352 (glutaconyl-CoA/methylmalonyl-CoA decarboxylase subunit gamma, orange), K23351 (glutaconyl-CoA/methylmalonyl-CoA decarboxylase subunit delta, lime), and K20509 (carboxybiotin decarboxylase, light blue). The other two most conserved flanking genes have best hits to K01604 (methylmalonyl-CoA decarboxylase subunit alpha, salmon) and K01571 (oxaloacetate decarboxylase, steel blue), but are below both thresholds.

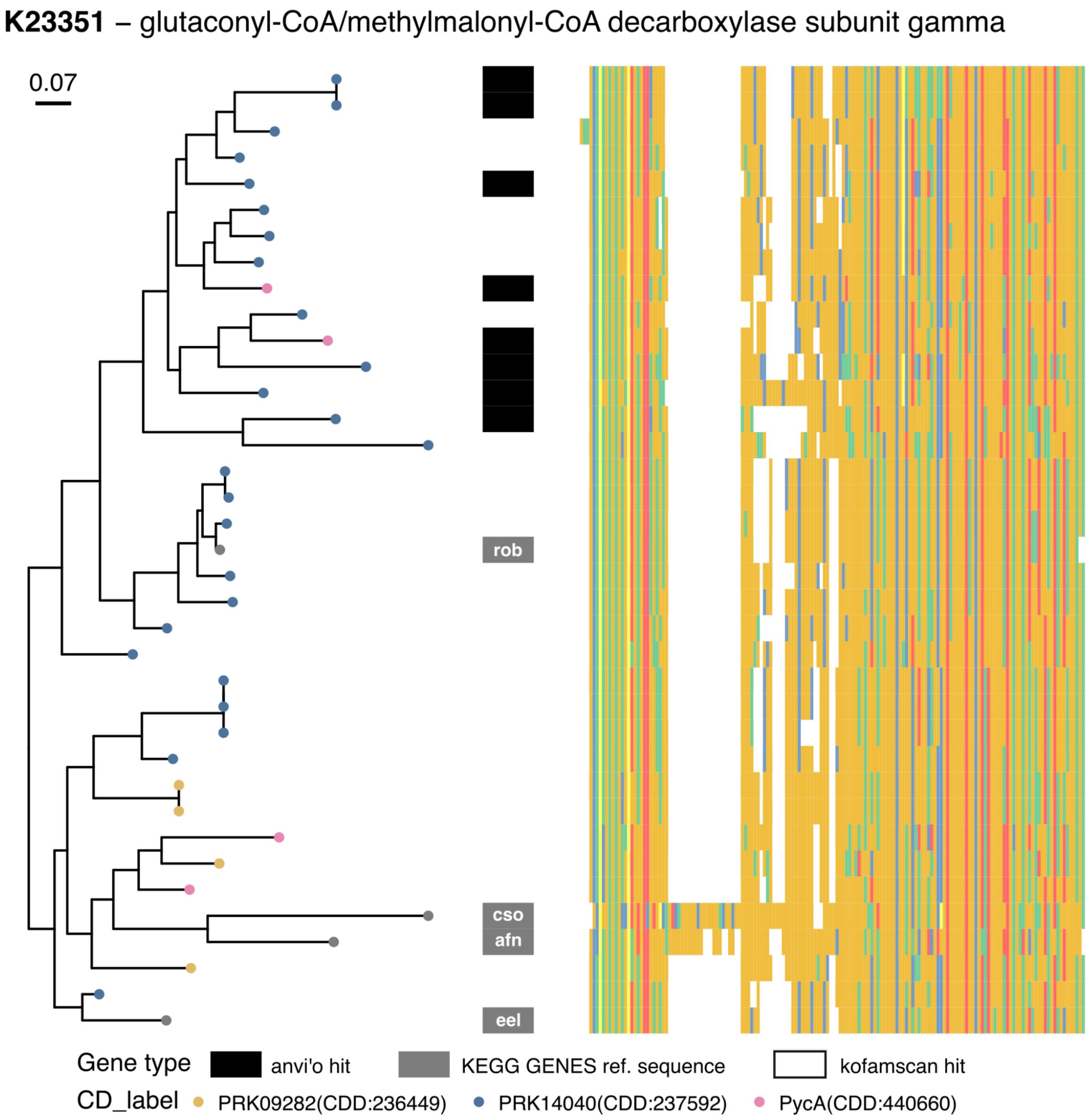

Supplementary Figure 12. Gene tree with scale bar and alignment of anvi'o and Kofamscan hits to the KO K23351 (glutaconyl-CoA decarboxylase gamma subunit). Selected reference sequences from the KEGG GENES database have been added in gray and labeled with the organism they originate in ("cso": *Lacrimispora saccharolytica*; "afn": *Acidaminococcus fermentans*; "rob": *Blautia obeum*; "eel": *Lachnospira eligens*). Genes matched by anvi'o only are marked in black; unmarked genes were found by both anvi'o and Kofamscan.

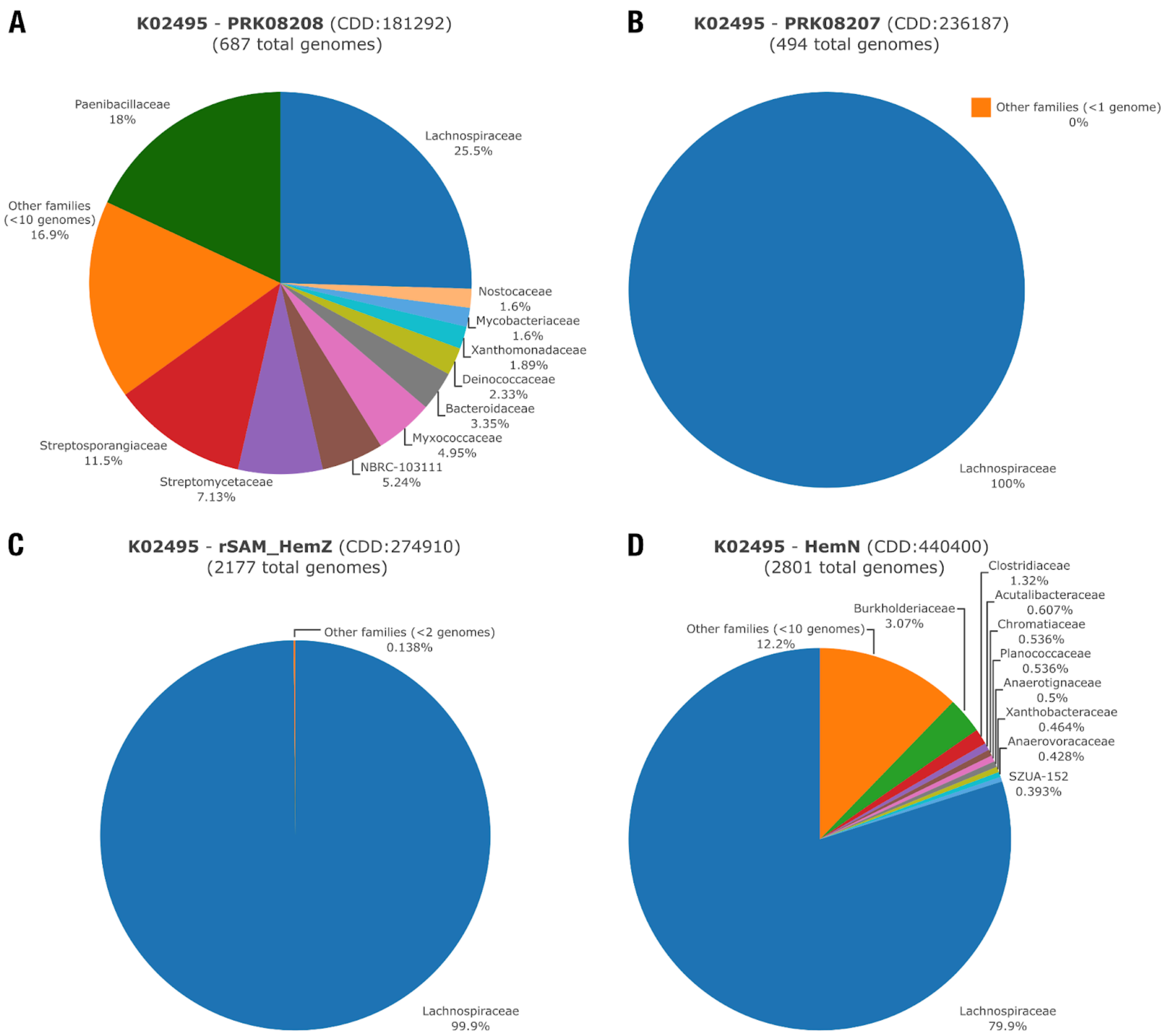

Supplementary Figure 13. Proportion of GTDB (v214) genomes with BLAST hits from *Lachnospiraceae* sequences annotated with K02495 in bacterial or archaeal families, split by their best protein family match in the NCBI Conserved Domain Database (CDD). **(A)** shows the taxonomic distribution of genomes with hits from PRK08208 (CDD: 181292) sequences, **(B)** shows PRK08207 (CDD: 236187) sequences, **(B)** shows rSAM_HemZ (CDD: 274910) sequences, and **(B)** shows HemN (CDD: 440400) sequences. Families with very few included genomes have been consolidated into the ‘Other families’ category.

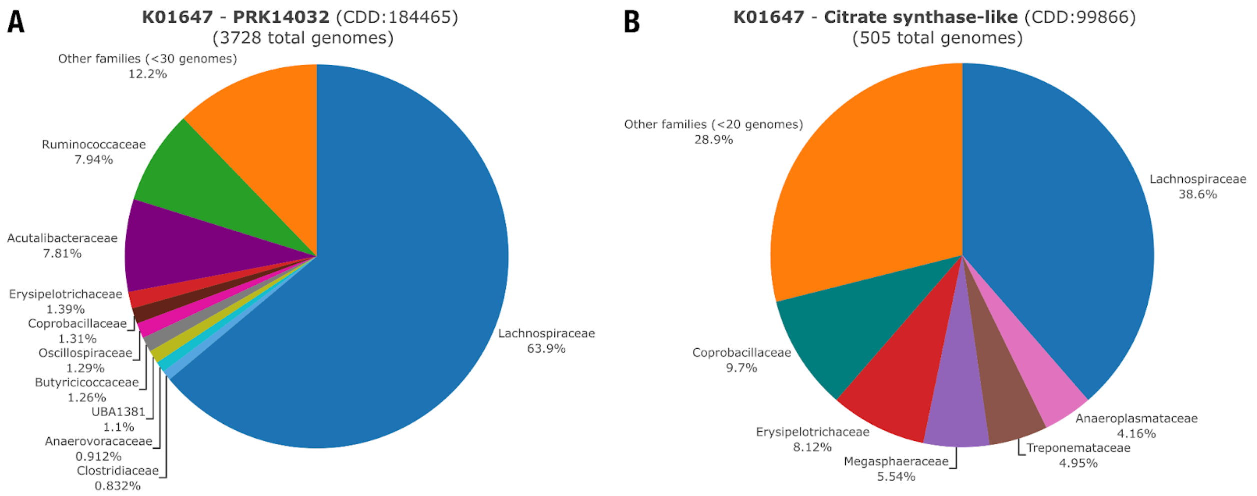

Supplementary Figure 14. Proportion of GTDB (v214) genomes with BLAST hits from *Lachnospiraceae* sequences annotated with K01647 in bacterial or archaeal families, split by their best protein family match in the NCBI Conserved Domain Database (CDD). **(A)** shows the taxonomic distribution of genomes with hits from PRK14032 (CDD: 184465) sequences, while **(B)** shows the distribution of genomes with hits from citrate-synthase-like (CDD: 99866) sequences. Families with very few included genomes have been consolidated into the 'Other families' category.

Supplementary Tables

Supplementary Table 1. Describes the genomes used in our analysis and the following information for each genome: assigned taxonomic family and species, number of gene calls, number of genes annotated per method, and mean module completeness per method. The method includes both which tool was run ('kofamscan'; 'microbeannotator'; 'anvio') and with which parameters ('default': default parameters for all three tools; 'refined': non-default parameters for both Kofamscan and MicrobeAnnotator; 'noheuristic': with the `--skip-bitscore-heuristic` flag for anvi'o; 'stray': with the `--include-stray-KOs` flag for anvi'o).

Supplementary Table 2. The mean genome size (nucleotide) of the samples in a family.

Supplementary Table 3. Number of gene annotations from each tool for the KEGG Orthologs belonging to the example modules in Figure 3 and Supplementary Figure 5.

Supplementary Table 4. Top 10 most dissimilar modules (highest absolute difference) based on average completeness scores averaged across per family for anvi'o and MicrobeAnnotator. Results from MicrobeAnnotator were added for comparison.

Supplementary Table 5. Percent of modules per family whose completeness scores increased using anvi'o or MicrobeAnnotator.

Supplementary Table 6. Per-pathway completeness scores in the 36 *Lachnospiraceae* genomes for the three custom butyrate biosynthesis pathway modules ('SUBSPEC': defined with substrate-specific enzymes whenever possible; 'BUCASYNOP': the 5-step core operon for butyryl-CoA synthesis; 'BUTANOATE': the full set of enzymes with all potential alternative KOs). The first column indicates the genome and subsequent columns indicate the module completeness scores as computed from the KOfams annotated by each tool (with default parameters).

Supplementary Table 7. Number of GTDB genomes with at least one BLAST hit from Lachnospiraceae sequences annotated with (a) K01647 or (b) K02495 in each microbial family. Hits were counted separately for each subgroup of query sequences with different CDD annotations – for K01647, these subgroups are PRK14032 (CDD: 184465) and citrate-synthase-like (CDD: 99866) sequences, while for K02495 there are four CDD subgroups: 181292, 236187, 274910, and 440400.

Supplementary Table 8. Top CDD hits for HemNZ subfamily sequences in Cheng et al. [14] Subfamilies are labeled as "Archaeal", "ChuW", "HemN", "HemW", "HemZ", or "RSM" (radical SAM methylase) as per the publication. The total hits per subfamily and per CDD accession are also reported, along with the CDD name and description, if available.

Supplementary Table 9. Top 10 and bottom 10 KOfams correlated with K01647, K01643, or K05942. Pearson correlation, p-values, q-values, and descriptions of each KOfam are reported. The descriptions of the three query KOfams themselves are also retained for readability.
